# The Impact of Type VI Secretion System, Bacteriocins and Antibiotics on Bacterial Competition of *Pectobacterium carotovorum* subsp. *brasiliense* and the Regulation of Carbapenem Biosynthesis by Iron and the Ferric-Uptake Regulator

**DOI:** 10.3389/fmicb.2019.02379

**Published:** 2019-10-18

**Authors:** Divine Yufetar Shyntum, Ntombikayise Precious Nkomo, Ntwanano Luann Shingange, Alessandro Rino Gricia, Daniel Bellieny-Rabelo, Lucy Novungayo Moleleki

**Affiliations:** ^1^Department of Biochemistry, Genetics and Microbiology, University of Pretoria, Pretoria, South Africa; ^2^Forestry, Agriculture and Biotechnology Institute, University of Pretoria, Pretoria, South Africa

**Keywords:** *Enterobacteriaceae*, type VI secretion system, Fur, carbapenem, *Pectobacterium brasiliense*, bacterial competition, potato microbiome, microbial interactions

## Abstract

The complexity of plant microbial communities provides a rich model for investigating biochemical and regulatory strategies involved in interbacterial competition. Within these niches, the soft rot *Enterobacteriaceae* (SRE) represents an emerging group of plant–pathogens causing soft rot/blackleg diseases resulting in economic losses worldwide in a variety of crops. A preliminary screening using next-generation sequencing of 16S rRNA comparatively analyzing healthy and diseased potato tubers, identified several taxa from *Proteobacteria* to *Firmicutes* as potential potato endophytes/plant pathogens. Subsequent to this, a range of molecular and computational techniques were used to determine the contribution of antimicrobial factors such as bacteriocins, carbapenem and type VI secretion system (T6SS), found in an aggressive SRE (*Pectobacterium carotovorum* subsp. *brasiliense* strain PBR1692 – *Pcb*1692) against these endophytes/plant pathogens. The results showed growth inhibition of several *Proteobacteria* by *Pcb*1692 depends either on carbapenem or pyocin production. Whereas for targeted *Firmicutes*, only the *Pcb*1692 pyocin seems to play a role in growth inhibition. Furthermore, production of carbapenem by *Pcb*1692 was observably dependent on the presence of environmental iron and oxygen. Additionally, upon deletion of *fur*, *sly*A and *exp*I regulators, carbapenem production ceased, implying a complex regulatory mechanism involving these three genes. Finally, the results demonstrated that although T6SS confers no relevant advantage during *in vitro* competition, a significant attenuation in competition by the mutant strain lacking a functional T6SS was observed *in planta*.

**IMPORTANCE:**

Soft rot *Enterobacteriaceae* (SRE) represents important phytopathogens causing soft rot/blackleg diseases in a variety of crops leading to huge economic losses worldwide. These pathogens have been isolated alongside other bacteria from different environments such as potato tubers, stems, roots and from the soil. In these environments, SREs coexist with other bacteria where they have to compete for scarce nutrients and other resources. In this report, we show that *Pectobacterium carotovorum* subsp. *brasiliense* strain PBR1692 – *Pcb*1692, which represents one of the SREs, inhibits growth of several different bacteria by producing different antimicrobial compounds. These antimicrobial compounds can be secreted inside or outside the plant host, allowing *Pcb*1692 to effectively colonize different types of ecological niches. By analyzing the genome sequences of several SREs, we show that other SREs likely deploy similar antimicrobials to target other bacteria.

## Introduction

For phytopathogenic bacteria, more is known about interactions with their hosts and virulence factors recruited to enforce successful colonization while relatively less is known about microbe–microbe interactions and how such interactions impact on niche colonizations (Bhunia, 2018; Glazebrook and Roby, 2018; Rodriguez-Moreno et al., 2018; Xin et al., 2018). Studies on the mammalian gut microbiome, the advent of new sequencing technologies, availability of many ‘omics’ data sets such as metagenomics, transcriptomics and proteomics are some of the factors that have brought microbial interactions to the fore (Knief et al., 2012; Yu and Zhang, 2012; Kõiv et al., 2015; Bost et al., 2018; Clayton et al., 2018). Thus, the past few decades have seen a rapid growth in the body of literature on microbial interactions.

Bacteria exist in complex multispecies communities which are mostly characterized by competition and to a lesser extent, cooperation (Stubbendieck and Straight, 2015; Bauer et al., 2018; García-Bayona and Comstock, 2018). In these interactions, survival depends on the ability to compete for resources in a given niche. Mechanisms of competition can be classified as exploitative or interference (Hibbing et al., 2010; Cornforth and Foster, 2013; Martin et al., 2017; Tyc et al., 2017; García-Bayona and Comstock, 2018). Exploitative competition is competition whereby microbes compete for scarce resources and the winner typically limits nutrient availability from competitors. Interference competition is characterized by production of specialized metabolites (antibacterial compounds). Microbes have a large arsenal of antimicrobial weapons that mediate competition and these include contact-dependent inhibition (CDI), such as the type VI secretion system (T6SS), antibiotic and/or bacteriocin production (Coulthurst et al., 2005; Aoki et al., 2010; Desriac et al., 2010; Russell et al., 2011, 2014; Grinter et al., 2012; Costa et al., 2015; Shyntum et al., 2015; Hachani et al., 2016; Wall, 2016; Verster et al., 2017; Bernal et al., 2018; Trunk et al., 2018). Over the years, many studies have interrogated mechanisms of the different antimicrobials ‘singularly’ while fewer studies have systematically investigated these factors in concert. For example, a growing number of studies have shown the role, mechanism of action and targets of T6SS, T5SS, and bacteriocins in bacterial competitions (Aoki et al., 2010; Bernal et al., 2018). There is therefore a need to study these systems collectively and under similar experimental conditions in order to gain better insight into their relative contribution to bacterial competition.

The T6SS is typically made up of 15–23 different proteins amongst which 13 proteins (TssA-M) are highly conserved in a wide range of bacteria and encode structural components of the T6SS (Mougous et al., 2006; Pukatzki et al., 2006). This secretion system has been extensively studied in several plant and animal pathogens and shown to secrete bactericidal effectors which inhibit growth of targeted bacteria lacking cognate immunity proteins (Poole et al., 2011; Bernal et al., 2018). The T6SS is regulated by quorum sensing, the ferric uptake regulator (Fur), histone-like nucleoid structuring protein (H-NS), RNA binding proteins (Hfq and RmsA) and several two-component systems (Silverman et al., 2012; Miyata et al., 2013; Salomon et al., 2014). In addition to the T6SS, *Pectobacterium* spp., upon induction with DNA damaging agents, secrete low molecular weight bacteriocins such as carocin (S1K, S2, and D), pectocin (P, M1, and M2) and the phage-like bacteriocin, carotovoricin (Ctv) (Chuang et al., 2007; Chan et al., 2009, 2011; Roh et al., 2010; Grinter et al., 2012). Ctv is encoded by the *ctv* gene cluster, containing 18 to 23 different genes encoding the lysis cassette, tail sheath and tail fiber (Yamada et al., 2006). Similar to T6SS effectors, bacteriocins are bactericidal effectors which play a role in both inter and intraspecies competition. In addition to bacteriocins, some *Pectobacterium*, *Dickeya*, and *Serratia* spp. produce the β-lactam antibiotic, 1-carbapen-2-em-3-carboxylic acid (carbapenem), which is targeted against different bacteria species including strains closely related to the carbapenem-producing strain. Carbapenem is encoded by *car*A/B/C/D/E/F/G/H genes, with *car*F and *car*G encoding immunity proteins (CarF/G) (McGowan et al., 1995, 1997, 2005). The *car* gene cluster was also shown to be regulated by the quorum sensing regulator, CarR, the stress response regulator SlyA (Hor) and Hfq (McGowan et al., 1995; Wang et al., 2018).

Soft Rot *Enterobacteriaceae* (SRE), comprising of *Dickeya* and *Pectobacterium* spp. is a group of pathogens causing huge economic losses on potato production worldwide (Adeolu et al., 2016; Charkowski, 2018). In addition to potato, SREs also cause disease in several monocots and dicots including pepper, avocado, onion, sugar beet, cacti, chicory, wasabi, rice, cabbage, marigold, burdock, cucumber, cassava, lettuce, pumpkin, and many other plants (Ma et al., 2007). They cause blackleg and soft rot diseases in the field or during post-harvest storage, respectively. Decaying potato tubers are characterized by multispecies bacterial communities that are both Gram positive and negative (Kõiv et al., 2015; Charkowski, 2018). Furthermore, within the SRE, it is quite common to isolate bacteria from *Pectobacterium* spp. as well as *Dickeya* spp., the two primary genera within *Enterobacteriaceae* from a single infected plant (Kõiv et al., 2015). Of these, *Pectobacterium carotovorum* subsp. *brasiliense* is an emerging SRE with global distribution (van der Merwe et al., 2010; Panda et al., 2012; Onkendi and Moleleki, 2014; Moraes et al., 2017; Ma et al., 2018; Voronina et al., 2019). Previous studies have shown that *Pcb* strain PBR1692 (referred to henceforth as *Pcb*1692) is able to inhibit growth of *Pectobacterium carotovorum* subsp. *carotovorum* WPP14 (*Pcc*) and *P. atrosepticum* SCRI1043 suggesting that many more bacteria can be inhibited by *Pcb*1692 thus giving it a fitness advantage, in a given ecological niche (Marquez-Villavicencio et al., 2011; Durrant, 2016). In this study, we use a combination of metagenomics, *in silico* analyses and various *in vitro* and *in planta* competition assays to identify and characterize bacterial targets, and the distinctive roles played by the different antimicrobial produced by *Pcb*1692 in bacterial competition, their regulation and possible contribution toward adaptation to diverse ecological niches.

## Materials and Methods

### Strains and Growth Conditions

All bacterial strains used in this study are listed in Supplementary Table S1. Bacterial strains were cultured in either Luria-Bertani (LB) medium or M9 minimum medium (Miller, 1972) supplemented with 0.4% sugar (glucose, sucrose, or glycerol) and grown aerobically at 37 or 28°C with or without agitation, as required by the given experimental conditions. Growth medium was supplemented with either 100 μg/ml ampicillin, 50 μg/ml kanamycin, 15 μg/ml gentamycin, 15 μg/ml tetracycline, or 50 μg/ml chloramphenicol. Antibiotics were purchased from (Sigma-Aldrich).

### DNA Isolation, Miseq Illumina Sequencing, and Data Analysis

Six diseased (soft rot disease) and six healthy potato tuber were collected from Gauteng, North West and Limpopo Province in South Africa. Genomic DNA (gDNA) was extracted from 1 g of fresh tissue using the ZYMO RESEARCH Quick-gDNA MiniPrep kit, according to the manufacturer’s instruction. DNA from either healthy or diseased tubers was pooled and aliquots used for 16S rRNA PCR amplification and Illumina Miseq sequencing. The 16S rRNA PCR was performed to amplify v3–v4 variable regions using primers 419F (ACTCCTACGGGAGGCAGCAG) and 806R (GGACTACHVGGGTWTCTAAT) (Fadrosh et al., 2014). The PCR reaction mixture consisted of 2.5 mM dNTPs, 10 × DreamTaq buffer (supplemented with 20 mM MgCl_2_), 0.5 U DreamTaq Polymerase (Fermentas), 10 μM each forward and reverse primer, 100 ng DNA template and nuclease free water up to the final reaction volume of 25 μl. PCR amplification conditions were as follows: denaturation at 95°C for 3 min, followed by 35 cycles at 95°C 30 s, 58°C 45 s, 72°C 2 min and the final extension at 72°C for 5 min. The purified PCR products (260/280 ratio ≥ 1.8) were sent to the Agricultural Research Council (ARC) Biotechnology platform, Pretoria, South Africa for 16S metagenomics library preparation, modified with Illumina specific adapters (Klindworth et al., 2013). Thereafter, the pooled samples were subjected to Miseq Illumina sequencing. Paired-end reads were trimmed using Trimmomatic v0.38 (Bolger et al., 2014), allowing minimum nucleotide quality average of 30 per 5 bp window in the sequences. Illumina adapters were removed using Cutadapt v1.18 (Martin, 2011). Chloroplast sequences were filtered out based on sequence identity assessed through Blastn (Camacho et al., 2009) with 16S rRNA acquired from *Solanum tuberosum*. High-quality reads were then submitted to SILVAngs rRNA-based data analysis pipeline under default settings (Quast et al., 2012). A total of 327,032 and 475,616 paired-end reads from diseased and healthy potato, respectively, were processed. After the initial systematic quality trimming, the 63,841 and 68,015 remaining reads from diseased and healthy samples, respectively, were filtered for chloroplast sequences. A total of 12,379 and 35,526 chloroplast sequences were removed from diseased and healthy samples respectively, and the remaining high-quality reads were analyzed using the SILVAngs pipeline (Quast et al., 2012). Subsequently, a total of 35,177 and 805 reads respectively from diseased and healthy samples were successfully classified into bacterial taxa. The Krona package (Ondov et al., 2011) was used to generate radial visualization of the resulting taxonomic profiles.

### Plant Extracts

Potato tuber extracts were prepared as previously described, with slight modifications (Mattinen et al., 2007). In summary, five grams of surface sterilized potato tubers (flesh and peel) were ground in 100 ml of distilled water using a blender. Plant tissue was removed by repeated centrifugation at 5000 rpm for 30 min at 4°C. The clarified plant extract was filter sterilized twice using a 0.2 μm filters, and aliquots stored at −16°C. Iron was selectively removed from potato tuber extracts by adding 200 μM of 2,2′-dipyridyl (Sigma Aldrich) dissolved in chloroform in a 1:1 ratio. The resulting mixture was vortexed for 2 min and iron free supernatant collected by centrifugation at 5000 rpm for 10 min at 4°C. The M9 agar plates were then supplemented with a 1:10 dilution of either plant extracts or plant extracts pre-treated with 2,2′-dipyridyl.

### Orthology Predictions and Binding Sites Analyses

Orthologs of bacteriocins and *car* genes were predicted by using OrthoMCL (Li et al., 2003) pipeline to analyze complete protein datasets from 100 *Pectobacterium* and *Dickeya* strains obtained from the RefSeq database as previously described (Bellieny-Rabelo et al., 2019b). Upstream regions of *exp*R, *sly*A, *car*R and *car*A from *Pectobacterium betavasculorum* strain NCPPB2795, *Pectobacterium atrosepticum* strain ICMP19972, *Pectobacterium carotovorum* subsp. *carotovorum* strain NCPPB312 and *Pcb*1692 genomes were retrieved from the NCBI online database^1^. Then the respective sequences of those regions were scanned using FIMO software from MEME package (Bailey et al., 2006) for the presence of putative Fur binding sites. This analysis was conducted using all known Fur binding sites found in MEME databases for prokaryotes: Prodoric (release 8.9) (Munch et al., 2003), RegTransBase v4 (Kazakov et al., 2006), and CollectTF (Kilic et al., 2014).

### *In vitro* Bacterial Competition Assays

Inter and intra species bacterial competition assays were performed as previously described (Shyntum et al., 2015). The complete list of bacteria used in this assay is provided in Supplementary Table S1. In summary, targeted bacteria were transformed with plasmid pMP7605 conferring gentamycin resistance (Lagendijk et al., 2010). Overnight cultures of *Pcb*1692 and targeted bacteria were normalized to an OD_600_ = 0.1, mixed in a 1:1 ratio and 20 μl spotted on LB or M9 agar containing no antibiotics. When required, M9 was supplemented with 0.01, 1, 10, or 50 μM of ferric sulfate (FeSO_4_.7H_2_O), magnesium sulfate (MgSO_4_.7H_2_O), manganese chloride (MnCl_2_.4H_2_O), zinc sulfate (ZnSO_4_.7H_2_O), nickel sulfate (NiSO_4_), cobalt chloride (CoCl_2_.H_2_O) or copper chloride (CuCl.H_2_O). Experiments were performed in triplicates and repeated three independent times.

Bacterial spots were briefly air dried and allowed to grow for 16 h at 28°C. When required, bacterial competition assays were performed anaerobically by placing agar plated in an anaerobic jar (Merck) containing moistened Anaerocult A (Merck) which absorbs oxygen and a moistened blue Anaerotest strip (Merck) which turns white in the absence of oxygen. Anaerobic jars were then tightly sealed and incubated overnight at 4°C. Thereafter, overnight spots were scraped off, serially diluted and plated on LB agar supplemented with gentamycin (15 μg/ml). The effect of iron on bacterial competition was determined using the following agar plates: (1) LB agar, (2) M9 minimal agar, (3) M9 supplemented with FeSO_4_.7H_2_O (10 μM), (4) M9 supplemented with potato tuber extracts, (5) M9 supplemented with FeSO_4_.7H_2_O (10 μM) and potato tuber extracts, (6) M9 supplemented with potato tuber extracts pre-treated with the iron chelator 2,2′-dipyridyl (200 μM) and (7) complementation of plate 6 listed above by the addition FeSO_4_.7H_2_O (10 μM) to potato tubers pre-treated with the 2,2′-dipyridyl (200 μM). All assays were performed in triplicates and three independent times. The results are presented as CFU/ml of target bacteria.

### Generation of *Pectobacterium carotovorum* subsp. *brasiliense* Mutant Strains

The different *Pcb*1692 mutant strains were generated by site-directed mutagenesis using the lambda red recombination technique (Datsenko and Wanner, 2000). In summary, overlap extension polymerase chain reaction (PCR) was used to generate a gene knockout cassette by fusing the upstream and downstream regions flanking the targeted gene to the kanamycin resistance genes (Shyntum et al., 2015; Tanui et al., 2017). The fused PCR product was then electroporated into *Pcb*1692 harboring pKD20 and transformants were selected on nutrient agar supplemented with either 50 μg/ml kanamycin or 50 μg/ml chloramphenicol. The list of primers used in this study is provided in Supplementary Table S2. The integrity of each *Pcb*1692 mutant strain was confirmed by PCR analyses, nucleotide sequencing and Southern blot analysis (results not shown). The list of mutant strains generated in this study is provided in Supplementary Table S3.

### Complementation of the *Pcb*1692 Mutant Strains

The list of primers used in this study is provided in Supplementary Table S2. The *Pcb*1692 *fur*, *exp*I, *PCBA_RS02805* (pyocin, designated *pyo*), *PCBA_RS02805:PCBA_RS02810* (pyocin and immunity genes, designated *pyo*I) and the T6SS genes *tss*A/B including their putative promoter sequences were PCR amplified and individually cloned into CloneJet 1.3 to generate p*fur*, p*exp*I, p*pyo*, p*pyo*I, and pT6, respectively. The tetracycline resistance gene (tet^*r*^) with its downstream promoter sequence was amplified from plasmid pME6031 (Heeb et al., 2000) with primers tetF/tetR. The ampicillin resistance gene located in plasmids p*fur*, p*exp*I, p*pyo*, p*pyo*I, pT6, and pJET-*sly*A (Bellieny-Rabelo et al., 2019a) was removed by inverse PCR, using primers IrF/IrR. The linearized vectors were then ligated to tet^*r*^ to generate pJET3-*fur*, pJET3-*sly*A and pJET3-*exp*I, pJET3-*pyo*, pJET3-*pyo*I, and pJET3-T6. The *Pcb*1692 *fur* promoter sequence was amplified with FurF/R and ligated to cloneJet 1.3 to generate p*fur-*1. The amp^*r*^ gene in p*fur-*1 was replaced with tet^*r*^, as previously described, generating pJET4. The promoterless *Pcb*1692 *car*C and *car*FG genes were amplified by PCR, followed by restriction digested with *Spe*I and cloned into pJET4 previously digested with *Spe*I, to generate pJET4-*car*C and pJET4-*car*FG, respectively. Plasmids pJET3-*fur*, pJET3-*sly*A, pJET3-*exp*I, pJET4-*car*C, pJET3-*pyo*, pJET3-*pyoI*, and pJET3-T6 were transformed into their corresponding mutant strains to generate the complemented mutant strains, *Pcb*1692Δ*fur*p*fur, Pcb*1692Δ*sly*p*sly*A, *Pcb*1692Δ*exp*Ip*exp*I, *Pcb1*692Δ*car*Cp*car*C, *Pcb*1692Δ*pyo*p*pyo*, *Pcb1*692Δ*pyo*Ip*pyo*I, and *Pcb1*692ΔT6pT6. Plasmid pJET4-*car*FG was transformed into *D. dadantii* in order to experimentally determine if the CarF and CarG proteins confer immunity to the carbapenem produced by *Pcb*1692. All transformants were maintained in LB supplemented with 15 μg/ml tetracycline.

### *In planta* Competition Assay

*In planta* competition assays were performed as previously described (Marquez-Villavicencio et al., 2011) with slight modifications. Targeted bacteria (*D. chrysanthemi, D. dadantii, Pcc, P. atrosepticum* and *Pcb*G4P5 were transformed with plasmid pMP7605 conferring gentamycin resistance (Lagendijk et al., 2010), as previously described. Wild-type *Pcb*1692, *Pcb*1692ΔT6, and *Pcb*1692ΔT6pT6 strains were grown overnight in LB supplemented with appropriate antibiotics. The optical density of overnight bacterial cultures was adjusted to OD_600_ = 1, resuspended in 1 X PBS, mixed in a 1:1 ratio and inoculated into surface sterilized potato tubers (cv. Mondial). Inoculated tubers were placed in moist plastic containers, sealed, and incubated for 72 h at 25°C. Macerated tuber tissue was scooped out and the CFU/ml of surviving targeted bacteria determined by serial dilutions on LB supplemented with gentamycin (15 μg/ml). The assays were performed three independent times each consisting of three technical repeats.

### Detection of Carbapenem and Bacteriocin Production by *Pcb*1692

Carbapenem production was determined by spot-on-lawn assays. Carbapenem production was determined for the following strains: wild-type *Pcb*1692, *Pcb*1692 mutant strains, HPI01 and laboratory stocks of *Pcb* strains whose genome sequences have not been determined (strains CC1, CC2, XT3, XT10, 358, G4P5, and G4P7). In summary, a 10 μl culture of *Pcb* strains, at OD_600_ = 0.1 was spotted on a lawn of freshly prepared targeted bacteria and a clear zone around *Pcb* was indicative of carbapenem production. To determine the role of iron in carbapenem production, lawns of targeted bacteria were prepared in M9 or M9 supplemented with iron (10 μM). Cultures of *Pcb* strains, OD_600_ = 0.1 were then spotted onto the M9 and production of a clear zone determined 24 h post-inoculation at 28°C. Bacteriocin production was determined by spot-on-lawn overlay method, as previously described (Roh et al., 2009). In summary, 10 μl of overnight cultures of *Pcb* strains were spotted on LB or M9, air dried and incubated overnight. Thereafter, bacteriocin production was induced by either UV irradiation or mitomycin C treatment. For mitomycin C induction, 10 μl of mitomycin C (50 μg/ml) was inoculated on top of *Pcb* strains, air dried and incubated in the dark for 5 h. For UV irradiation, cultures of *Pcb* strains were exposed to UV light for 10 s and incubated in the dark for 5 h. Thereafter, bacteriocin-induced *Pcb* cultures were killed with chloroform fumes for 10 min and then air dried for 30 min. Twenty microliters of overnight cultures of targeted bacteria was inoculated into 25 ml of cooled 0.7% agar and overlayed on bacteriocin induced *Pcb*1692 strains. Clear zones around *Pcb* strains were indicative of bacteriocin production. In control experiments, *Pcb* strains were not exposed to UV or mitomycin C. All experiments were repeated three times. To determine the ability of *Pcb*1692 strains to produce carbapenem and bacteriocins under anaerobic conditions, the experimental procedures were the same as above except that bacteria were grown anaerobically in anaerobic jars (Merck) containing moistened Anaerocult A (Merck) which absorbs oxygen and moistened blue Anaerotest strips (Merck) which turns white in the absence of oxygen. Anaerobic jars were then tightly sealed and incubated overnight.

### Statistical Analysis

All experiments were performed in triplicates and three independent times. Where applicable, an unpaired, two-tailed Student’s *t*-test was performed to determine statistical significance and a *p*-value less than 0.05 (*p* < 0.05) was considered to be a statistically significant difference.

## Results

### The Microbiome of Potato Tubers

**FIGURE 1:**
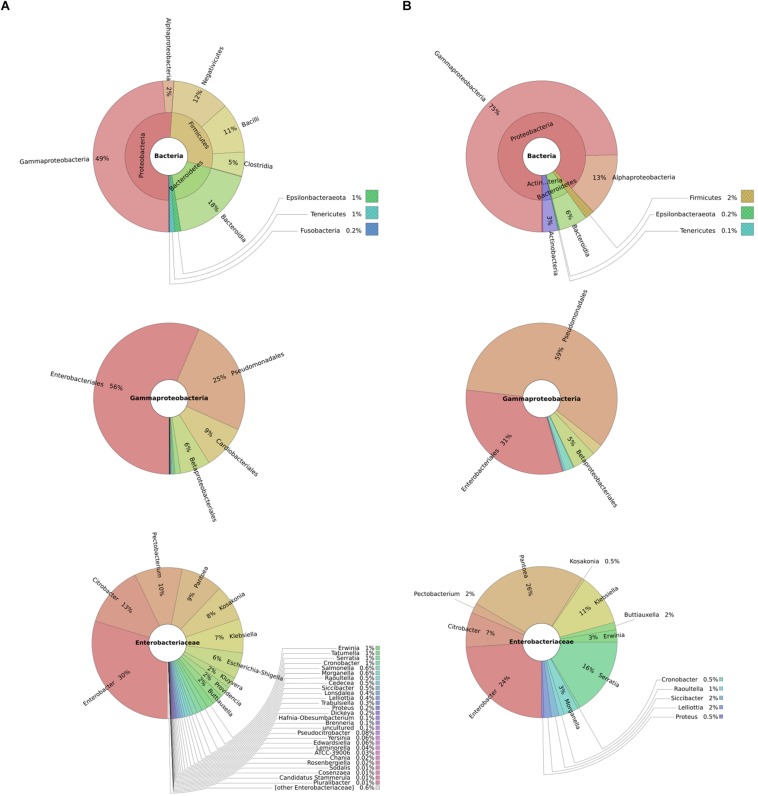
Taxonomic profile of diseased potatoes assessed through shotgun sequencing: metagenomics analyses represented by composite pie-charts depict the taxonomic representation obtained from sequenced dataset. For each graph, outer radiuses represent the relative proportion of reads assigned to different taxa of the inner circle taxon. Charts under column **(A)** represents the relative proportion of reads found in diseased potato tubers while **(B)** represents reads from healthy potato tubers.

To understand typical microbial flora on potato tubers and thus infer potential competitors, we performed 16S rRNA metagenomics of diseased and healthy potato tuber tissues. We found overall a high representation of bacteria from the phylum *Proteobacteria* in both diseased (51%) and healthy potato tubers (75%). On the other hand, *Firmicutes* made up 2 and 28% of bacteria in healthy and diseased potato tubers, respectively, implying an increasing population of *Firmicutes* as tubers progress from a healthy to a diseased state. In both diseased and healthy tissues, the dominant orders within the *Gammaproteobacteria* were *Enterobacteriales* (56 and 31%, respectively) and *Pseudomonadales* (25 and 59%, respectively) (Figure 1). Within *Enterobacteriales*, the analyses identified the following genera: *Pantoea, Enterobacter, Serratia* together with genera such as *Yersinia, Klebsiella, Salmonella*, *Escherichia*, *Citrobacter*, and *Shigella* in both healthy and diseased potato (Figure 1). As can be expected, soft rot bacteria within the *Pectobacterium* and *Dickeya* genera were well-represented in diseased potato (Figure 1).

### Bacterial Growth Inhibition by *Pcb*1692 Is Associated With Inter and Intra Species Competition

**FIGURE 2:**
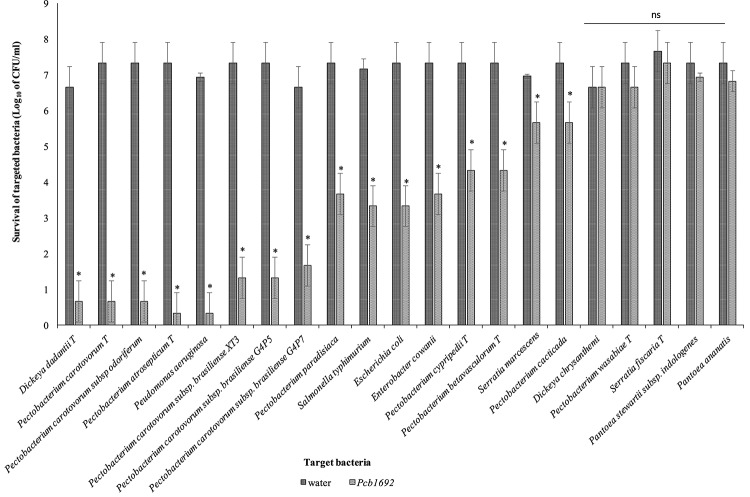
Interbacterial competition between *Pectobacterium carotovorum* subsp. *brasiliense* (*Pcb*1692) and targeted bacteria: *Pcb*1692 and targeted bacteria were co-cultured in a 1:1 ratio on LB and the log_10_ CFU/ml of surviving targeted bacteria determined 24 h post-incubation. Experiments were performed in triplicates and repeated three independent times. Bars represent mean values, error bars represent one standard deviation and asterisk represent *p*-values for the differences in CFU/ml of recovered target bacteria when co-cultured with *Pcb*1692 relative to water control as determined by the two-tailed Student’s *t*-test. *p* < 0.05 was considered to be statistically significant. P, *Pectobacterium*.

*Pcb*1692 has previously been shown to inhibit growth of some closely related SREs such as *Pcc* and *D. dadantii* (Marquez-Villavicencio et al., 2011; Durrant, 2016). In this study, we wanted to determine the spectrum of bacteria killed by *Pcb*1692 in competition assays. We hypothesized that *Pcb* strains will likely compete with bacteria that they typically share the same niche. We used the metagenomics data to identify bacteria (at genus level) generally found in both healthy and diseased potato tubers. We combined the metagenomics data analysis with literature searches to identify bacteria (genus and species) previously isolated from diseased potato tubers (Hollis, 1951; Bulgarelli et al., 2012; Gupta et al., 2014; Schreiter et al., 2014; Donn et al., 2015; Hong et al., 2015; Kõiv et al., 2015). With this combined information, we selected a number of different bacteria for subsequent competition assays. We chose to use type strains as they have either draft or full genome sequences publicly available. This choice was necessitated by the need to perform *in silico* analyses in order to understand the bases of susceptibility or resistance of target strains, to killing by *Pcb*1692. Target cells were co-cultured in water or with *Pcb*1692. As has been shown by others, the results show that *Pcb*1692 can inhibit growth of closely related *Pectobacterium* and *Dickeya* species when co-cultured in LB agar (Figure 2). The magnitude of this inhibition is strain specific. For example, up to a 6-log reduction in CFU/ml (*p* < 0.01) was observed when *Pcb*1692 was co-cultured with some members of SRE within the phylum *Proteobacteria*, such as *P. atrosepticum*, *Pcc*, *Pectobacterium carotovorum* subsp. *odoriferum* (*Pco*), and *D. dadantii* while a 1–3 log reduction (*p* < 0.05) was observed for *P. paradisiaca, P. cipripedii, P. cacticida* and *P. betavasculorum.* The study also demonstrates that within *Proteobacteria*, *Pcb*1692’s competitive ability is not only restricted to SREs. Our results show that *Pcb*1692 can inhibit other members of *Enterobacteriaceae* such as *Enterobacter cowanii, Salmonella typhimurium, Escherichia coli* and *Serratia marcescens* and one *Pseudomonad* (*Pseudomonas aeruginosa*) (Figure 2). The ability of *Pcb* to inhibit *P. aeruginosa, S. typhimurium, E. coli, E. cowanii* and *S. marcescens*, which have previously been isolated from potato tubers (Malek et al., 2013; Kõiv et al., 2015) likely gives *Pcb* a fitness advantage in potato tubers, however, this needs to be experimentally verified. Interestingly, the results also showed that *Pcb*1692 was able to significantly inhibit growth of other closely related *Pcb* strains such as G4P5, G4P7, XT3, and XT10 (5 log reduction in CFU/ml *p* < 0.01) but not strains HPI01, 358, CCI, and CC2 (Figure 2 and Supplementary Table S4). The intra and interspecies inhibition observed could either be due to nutrient competition, phage induction, small metabolite production, or secretion of effectors by *Pcb*1692 for which closely related strains lack the cognate immunity factor, a premise that is supported by our subsequent *in silico* analysis. In addition, the data also showed that *Pcb*1692 was unable to inhibit *in vitro* growth of other *Proteobacteria* such as *D. chrysanthemi, P. wasabiae*, *S. ficaria, Pantoea ananatis*, *Pantoea stewartii* subsp. *indologenes*, and *Firmicutes* such as *Bacillus subtilis* and *B. cereus* in rich LB medium. Together, these findings demonstrate that *Pcb*1692 readily inhibits growth of both (1) most SREs analyzed in this study, including closely related *Pcb* strains, as well as (2) non-SREs.

### Iron in Potato Tuber Extracts Plays an Essential Role in Bacterial Competition

**FIGURE 3:**
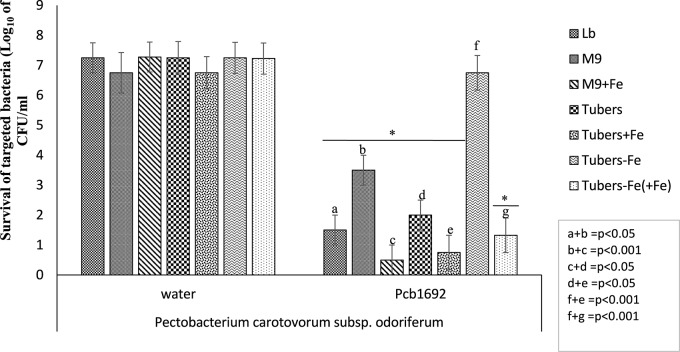
Effect of iron in interbacterial competition: *Pectobacterium carotovorum* subsp. *brasiliense* 1692 and targeted bacteria were co-cultured in a 1:1 ratio in either M9, M9 supplemented with either potato tuber extracts or 10 μM ferric iron. The iron in plant extracts was removed using 2,2′-dipyridyl. Results are presented as the CFU/ml of recovered targeted bacteria following 24 h co-culture. Asterisk indicates statistically significant differences *p* < 0.05 in the recovery of targeted bacteria co-cultured with *Pcb*1692 relative to water control as determined by two-tailed Student’s *t*-test. *Pco*, *Pectobacterium carotovorum* subsp. *odoriferum*. Experiments were performed in triplicates and repeated three independent times. “a–g” represent the different competition conditions used in this study and corresponds to the pattern fill shown in the key LB, M9, M9+Fe, Tuber, Tuber+Fe, Tuber-Fe, and tuber-Fe(+Fe).

Potato tubers contain several metals such as iron, potassium, zinc, copper, manganese, lead, cadmium; however, these metals are sequestered in different plant compartments and not readily available to bacteria in the intracellular spaces (Luis et al., 2011; Tack, 2014). Some of these metals play a role in virulence and bacterial competition, by either acting as enzyme co-factors or co-activator/-repressors of some transcriptional regulators (Fones and Preston, 2013). We therefore investigated the effect of some of these metals on the observed ability of *Pcb*1692 to kill targeted bacteria *in vitro*. For this part of the study, *Pcb*1692 was co-cultured with *Pcc* and *D. dadantii* in M9 minimal media supplemented with different metals at concentrations 0.01 to 50 μM, representing physiological levels typically found in potato tubers (Luis et al., 2011; Tack, 2014) (see section “Materials and Methods”). The results showed that while low concentrations of copper and cobalt (0.01 and 1 μM) had no effect on competition, higher concentrations of both metals (10 and 50 μM) inhibited growth of both *Pcb*1692 and *Pcc* (Supplementary Data Sheet S1). In addition, different concentrations of magnesium, manganese, zinc, nickel (0.01, 1, 10, and 50 μM) had no effect on bacterial competition (Supplementary Data Sheet S1). On the other hand, the addition of ferric iron (10 or 50 μM) to M9 minimal medium enhanced the ability of *Pcb*1692 to kill either *Pcc*, *Pco*, or *D. dadantii* while lower concentrations (0.1 and 1 μM) had no effect on bacteria competition (Figure 3 and Supplementary Data Sheet S2). Therefore, 10 μM ferric iron was used to supplement M9 medium in all downstream assays associated with iron. For competition assays, target bacteria were co-cultured in water (controls) or with *Pcb*1692 (Figure 3). Figure 3 shows that when *Pco* was co-cultured with *Pcb*1692 in LB (rich) medium, fewer cells of target bacteria were recovered compared to controls (*p* < 0.05). However, a 1–2 log increase in the number of target bacteria cells recovered after co-culture in nutrient-poor M9 medium (relative to rich medium) was observed. This indicated a statistically significant reduction (*p* < 0.05) in the ability of *Pcb*1692 to kill targeted bacteria in nutrient-poor medium compared to LB rich medium. The magnitude of inhibition by *Pcb*1692 significantly increased by 2 log when M9 agar was supplemented with potato tuber extracts. Thus, adding tuber extracts to minimal medium enriched the medium and hence a similar outcome on competition as with rich LB medium was observed. When M9 was supplemented with iron, fewer competitor/target cells were recovered (relative to rich medium), indicating a statistically significant (*p* < 0.05) increase in the *Pcb*1692 killing ability. Addition of the iron chelator 2,2′-dipyridyl to M9 tuber-supplemented medium eliminated ability of *Pcb*1692 to kill competing bacteria, while addition of 10 μM of ferric iron to M9 media restored competition in the absence of plant extracts. Together these findings demonstrate that the presence of iron increased the ability of *Pcb*1692 to inhibit targeted bacteria. In the next sections, we report the approaches used to establish whether or not the effect of iron is associated with the expression of T6SS, carbapenem or bacteriocin production in *Pcb*1692.

### *In silico* Identification of Genes Associated With Antibacterial Activity

A transcriptome profiling experiment performed previously in our laboratory, showed that *Pcb*1692 genes encoding the T6SS and bacteriocins (carotovoricin and pyocin) were up-regulated during *in planta* (potato tuber) infection (Bellieny-Rabelo et al., 2019b). The same study also showed that gene homologs of the *Pcb*1692 T6SS were found to be present in the genome sequences of all publicly available genome sequences of *Dickeya* and *Pectobacterium* (Bellieny-Rabelo et al., 2019b). Similarly, while homologous sequences of *Pcb*1692 carotovoricin gene cluster were present in all publicly available genome sequences of *Pectobacterium* spp. it is missing from the genome sequences of all *Dickeya* spp. except *D. dianthicola* RNS049 and *D. zeae* strains CSLRW192, EC1, and ZJU1202. The above analysis was extended to include orthology relationship between the *Pcb*1692 protein sequences associated with the carbapenem and the S-type pyocin in order to determine their presence or absence in other *Pectobacterium* and *Dickeya* spp.

**FIGURE 4:**
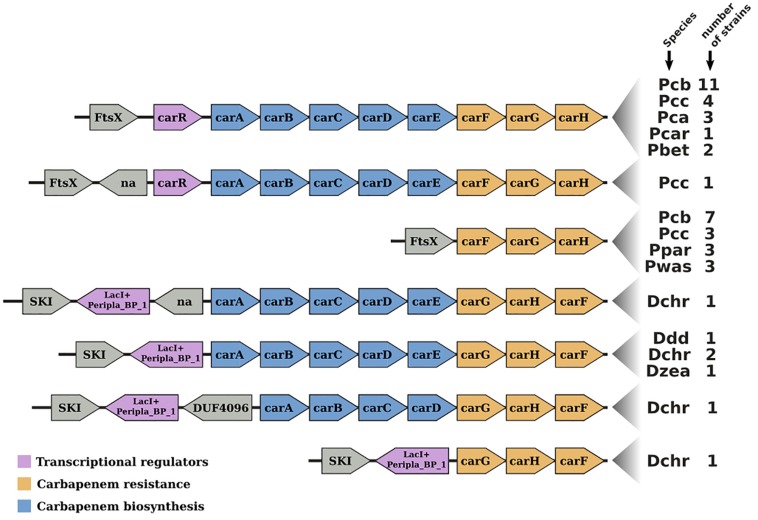
Gene-neighborhood screening of the carbapenem gene cluster (*car*) in Soft-Rott-*Enterobacteriaceae* (SRE): 100 SRE genomes were analyzed and all bacteria strains that conserve segments of the *car* cluster were screened. All known *car* genes are labeled in the figure. Genes unrelated with the *car* cluster are labeled using functional domains detected in the respective encoded proteins when applicable, otherwise these are labeled with “na.”

The *Pcb*1692 *car* gene cluster was found to contain all six conserved genes required for synthesis of carbapenem (*car*A/B/C/D/E/H) including genes encoding the carbapenem immunity proteins (CarF/G). Homologous *car* gene clusters were also identified in *P. betavasculorum* ATCC 43762 (T), *Pcc* ATCC 15713(T), *D. chrysanthemi* NCPPB 3533, *D. zeae* CSL RW192, *D. zeae* Ech1591, and several strains of *Pcb* (Figure 4). This gene cluster was found to be missing from all publicly available genome sequences of *P. wasabiae*, *P. atrosepticum*, *P. parmentieri*, *D. solani*, and *D. dadantii* (exempting *D. dadantii* subsp. *dieffenbachiae* NCPPB2976) (Sheet 1 in Supplementary Table S5). Similarly, the *car* gene cluster was found to be missing from the genome sequence of *Pcb* strains (BC1, YCD21, YCD49, YCD62, and YCD65). Interestingly, although lacking the *car* gene cluster, *P. wasabiae* strains (CFBP3304, NCPPB3701, and NCPPB3702), *P. parmentieri* strains (CFIA1002 and RNS08421a) and *Pcb* strains (BC1, YCD21, YCD49, YCD62, and YCD65) all retained the carbapenem immunity gene *car*F*/car*G, suggesting that they may be resistant to the carbapenem produced by some *Pectobacterium* and *Dickeya* spp.

Furthermore, we identified a putative S-type pyocin encoded by the *Pcb*1692 *PCBA_RS02805*. This protein (794 amino acids) was predicted to contain a colicin-like bacteriocin tRNase domain (Pfam: PF03515) (Bateman et al., 2004), suggesting it might kill other bacteria by degrading their tRNA. Homologs of the *Pcb*1692 pyocin were identified in the genome sequence of *Pcb* strains (LMG21371, HPI01, YCD29, ICMP19477, BC1), *Pcc* strains (PCC21, NCPPB3395, and YCD57) and *P. betavasculorum* NCPPB2795 but missing from all publicly available genome sequences of *P. atrosepticum*, *P. wasabiae*, and *Dickeya* (Sheet 2 in Supplementary Table S5). Downstream of the *Pcb*1692 *PCBA_RS02805* gene is *PCBA_RS02810* encoding the cognate S-pyocin immunity factor (∼90 amino acids), with homologs present in all SREs encoding S-pyocin. Interestingly, although the genome sequences of *Pco* strains (S6, S6-2, Q106, Q32, and Q3) and *Pcc* strains (Y57 and 3F-3) do not have genes encoding the homologous *Pcb*1692 S-pyocin, they encode S-pyocin immunity factors 100% identical to the one found in *Pcb*1692 (PCBA_RS02810). Overall, this analysis demonstrates that the *Pcb*1692 toxin/antitoxin modules analyzed in this study are not conserved in all bacteria including some strains of *Pcb* and may therefore be a determining factor in the outcome of inter or intra species competition among SRE.

### The *Pcb*1692 Carbapenem Gene Cluster Plays a Role in Bacterial Competition *in vitro*

**FIGURE 5:**
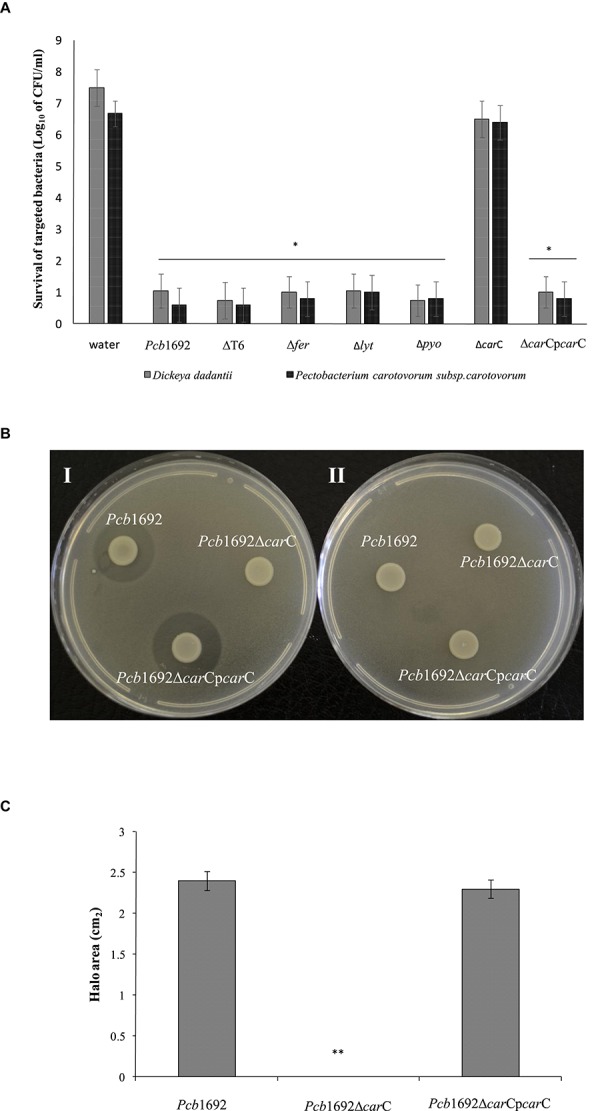
Interbacterial competition with *Pectobacterium carotovorum* subsp. *Brasiliense* 1692 mutant strains. **(A)** Wild-type *Pcb*1692 and its isogenic mutant (T6, *fer*, *lyt*, and *car*C) were co-cultured with *D. dadantii* and *Pcc* for 24 h. The data shows the log_10_ CFU/ml of recovered *D. dadantii* and *Pcc* following co-culture. The horizontal line with an asterisk represents the range of samples with statistically significant differences (*p* < 0.05) relative to water controls. **(B)** (i) Clearing zone due to carbapenem produced by strains of *Pcb*1692 spotted on a lawn of *D. dadantii* with circularize plasmid PJET4, (ii) No clearing zones when of *Pcb*1692 is spotted on a lawn of *D. dadantii* expressing *car*F and G resistance genes from plasmid pJET4-*car*FG **(C)** Halo area was measured using ImageJ and data presented as the mean, error bars represent one standard deviation and asterisk represent statistically significant difference (*p* < 0.05) as determined by the two-tailed Student’s *t*-test. Experiments were performed in triplicates and repeated three independent times.

**FIGURE 6:**
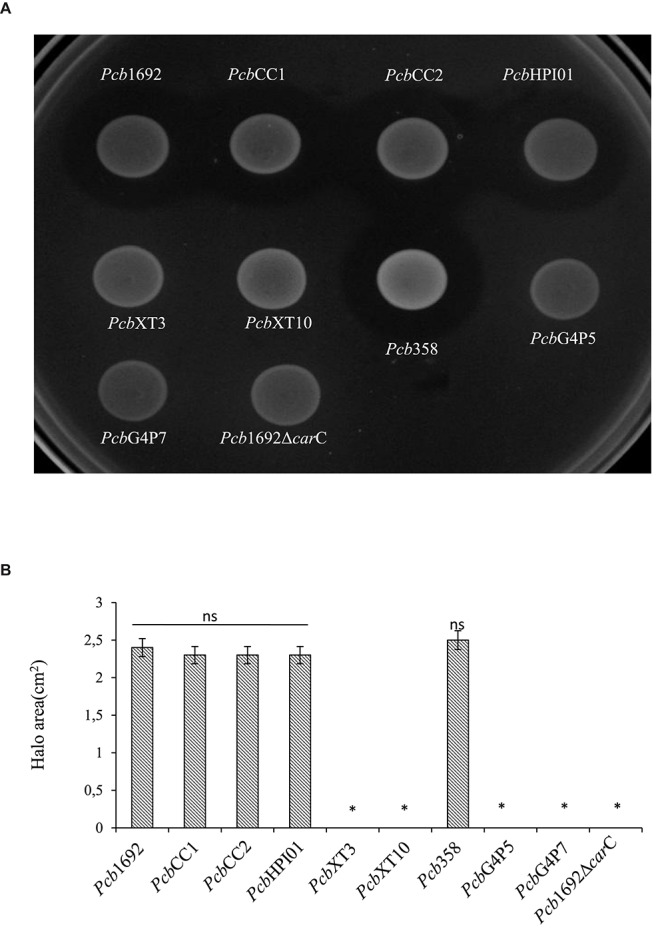
Carbapenem production by environmental strains of *Pectobacterium carotovorum* subsp. *brasiliense.* The ability of laboratory strains of *Pcb* isolated from South Africa to produce carbapenem when spotted on a lawn of *Dickeya dadantii* was determined by the spot-on-lawn assay. **(A)** The presence of a clear zone around the spotted *Pcb* was indicative of carbapenem production. *Pcb*1692 was used as a control given that this strain produces carbapenem. **(B)** Halo area was measured using ImageJ and data presented as the mean, error bars represent one standard deviation and asterisk represent statistically significant difference (*p* < 0.05) as determined by the two-tailed Student’s *t*-test. Experiments were performed in triplicates and repeated three independent times.

In order to investigate the relative contribution of the *Pcb*1692 T6SS, carotovoricin, carbapenem, and the putative S-type pyocin in competition, we generated *Pcb*1692 mutants with deletions in genes associated with the production of S-pyocin (*Pcb*1692Δ*pyo*), carbapenem (*Pcb*1692Δ*car*C), carotovoricin (*Pcb*1692Δ*lyt* and *Pcb*1692Δ*fer*) and biosynthesis of a functional T6SS (*Pcb*1692ΔT6). Targeted bacteria (*D. dadantii* or *Pcc*), earlier shown to be inhibited by wild-type *Pcb*1692, were co-cultured *in vitro* with either water or the different *Pcb*1692 mutant strains. The results show that the CFU/ml of recovered targeted bacteria following co-culture with the *Pcb*ΔT6, Δ*lyt*, Δ*fer*, and Δ*pyo* mutant strains was similar to the CFU/ml of recovered targeted bacteria following co-culture with the wild-type strain. However, the *Pcb*1692Δ*car*C mutant strain lost the ability to inhibit growth of *D. dadantii* and *Pcc* (Figures 5A,C). Trans-expression of the *car*C gene in *Pcb*1692Δ*car*C restored the ability of the complemented strain to inhibit targeted bacteria, similar to wild-type *Pcb*1692 (Figures 5A,C). Together, these findings suggest that carbapenem production by *Pcb*1692 is associated with inter and intrabacterial competition *in vitro*. Trans-expression of the *Pcb*1692 *car*F/G genes in *D. dadantii* prevented growth inhibition of this strain when co-cultured with wild-type *Pcb*1692 confirming that growth inhibition of these bacteria is associated with *Pcb*1692 carbapenem and that the *Pcb*1692 *car*F/G genes confer immunity to the *Pcb*1692 carbapenem (Figure 5B). In addition, our results showed that while *Pcb* strains CC1, CC2, HPI01, and 358 can produce carbapenem, which inhibit growth of *D. dadantii* (Figure 6), *Pcb* strains XT3, XT10, G4P5, and G4P7 do not produce carbapenem under the same experimental conditions (Supplementary Table S4). Similarly, the carbapenem produced by *Pcb* strains 1692, CC1, CC2, HPI01, and 358 was found to inhibit growth of *Pcb* strains XT3, XT10, G4P5 and G4P7, suggesting that the latter group of strains lack the cognate carbapenem immunity factor. Together, *in vitro* and *in silico* analyses uncover the contrasting conservation patterns of the carbapenem biosynthesis cluster in SREs, highlighting its critical role in interference competition among these organisms. With this information, we proceed toward assessing possible regulatory processes involved in the control of carbapenem biosynthesis.

### Iron Is Required for Carbapenem Production by *Pcb*1692 in Minimal Media and the Car Gene Cluster Is Regulated by Fur, SlyA, and ExpI

**FIGURE 7:**
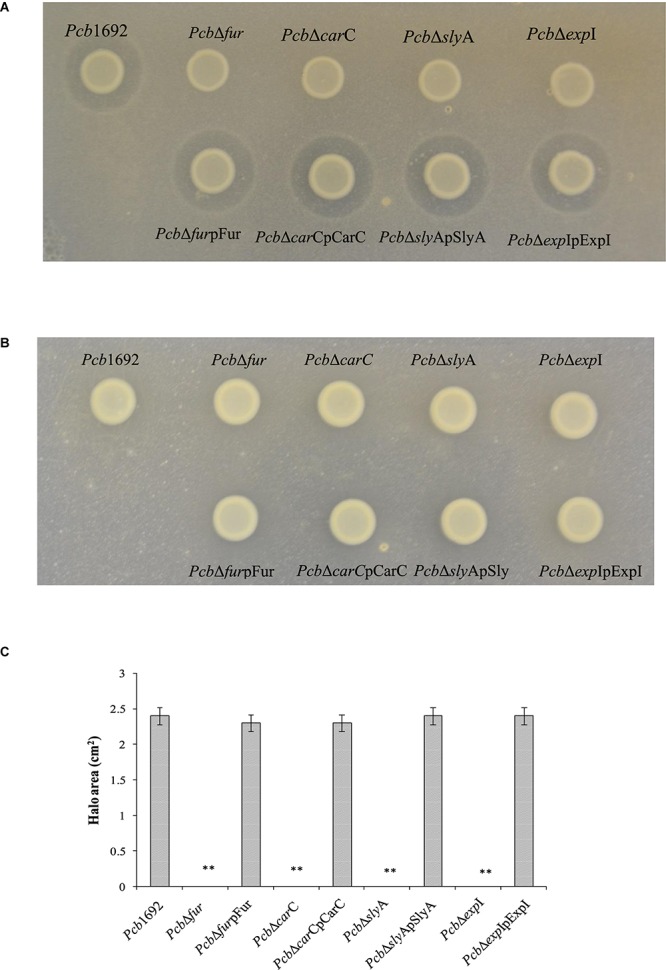
Iron is required for production of carbapenem by *Pectobacterium carotovorum* subsp. *brasiliense* 1692. *Pcb*1692 wild-type mutant and complemented mutant strains were spotted on a lawn of *P. atrosepticum* and clear zone around the spotted *Pcb* strains was indicative of carbapenem production. **(A)** Carbapenem production on LB agar by *Pcb* strains. **(B)** No detectable levels of carbapenem were observed on M9. **(C)** Halo area was measured using ImageJ and data presented as the mean, error bars represent one standard deviation and asterisk represent statistical significant difference (*p* < 0.05) as determined by the two-tailed Student’s *t*-test. Experiments were performed in triplicates and repeated three independent times.

Having demonstrated that the *Pcb*1692 carbapenem plays a role in *in vitro* interbacterial competition, we next investigated how this gene cluster is regulated and under which conditions *Pcb*1692 produces carbapenem. To this end, we included *Pcb*1692 transcriptional regulator mutant strains including the ferric uptake regulator (Tanui et al., 2017), *N*-acyl homoserine lactone synthase (ExpI) (Moleleki et al., 2017) and the stress response regulator SlyA. The results showed that while wild-type *Pcb*1692 produced a clear zone of inhibition when cultured on a lawn of *P. atrosepticum*, the *Pcb* mutant strains (Δ*car*C, Δ*sly*A, Δ*exp*I, and Δ*fur*) did not produce this clear zone associated with carbapenem production (Figures 7A,C). Loss of carbapenem production could be complemented by trans-expression of corresponding genes in the *Pcb*1692 mutant strains (Figures 7A,C). In addition, while wild-type *Pcb*1692 and the complemented mutant strains produced carbapenem on LB, they all lost the ability to produce carbapenem on M9 supplemented with 0.4% of glucose (Figure 7B). Interestingly, carbapenem production was restored when M9 was supplemented with 10 μM of ferric iron (Supplementary Data Sheet S3). These findings implicate iron and the iron homeostasis protein Fur in the regulation of carbapenem production in *Pcb*1692. Aiming to predict whether Fur is able to regulate carbapenem production in *Pcb*1692 directly, or indirectly, we performed *in silico* analysis using the MEME package (Bailey et al., 2006) to search for *fur* binding sites in the promoter regions of *car*R, *sly*A, *exp*I, and *exp*R. Interestingly, the analysis identified *fur* binding sites in the promoter sequences of *car*R, *sly*A, and *exp*R but not in the promoter sequences of *exp*I and *car*A (the first gene in the *car* gene cluster), however, this still needs to be experimentally verified (Supplementary Table S6). Given that CarR, and not ExpR, is required for carbapenem production (Rivet, 1998; Coulthurst et al., 2005; Hamed et al., 2013), the *Pcb*1692 Fur protein may be able to indirectly control the transcription of carbapenem production via *car*R, *sly*A, or both.

### The *Pcb*1692 T6SS Plays a Role in Interspecies but Not Intraspecies Competition *in planta*

**FIGURE 8:**
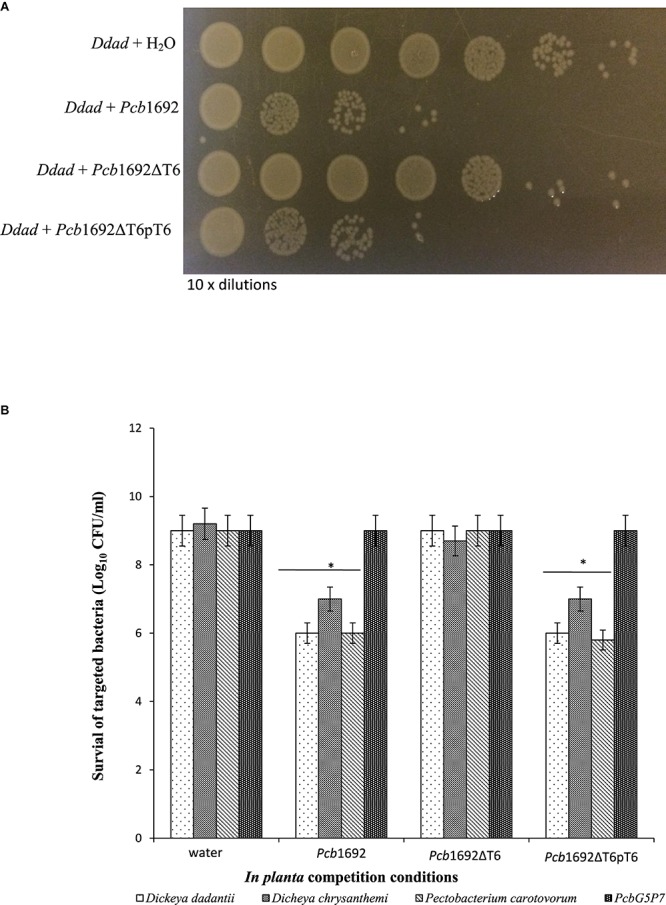
Effect of the T6SS in *Pcb*1692 anti-bacterial competition *in planta*. Gentamycin resistant targeted bacteria (*Dickeya dadantii, D. chrysanthemi*, and *Pcc*) were mixed in a 1:1 ratio with either water, wild-type *Pcb*1692, *Pcb*1692ΔT6 *or Pcb*1692ΔT6pT6, inoculated into potato tubers and incubated for 24 h and **(A)** survival of *D. dadantii* enumerated by 10-times serial dilution on gentamycin (15 μg/ml) agar. **(B)** Quantification of survival of targeted bacteria presented as log_10_CFU/ml. Asterisks indicated statistically significant differences (*p* < 0.05) relative to water control, as determined by the two-tailed Student’s *t*-test. Experiments were performed in triplicates and repeated three independent times.

Given the *Pcb*1692 T6SS did not appear to play a role in bacterial competition *in vitro*, but was upregulated in potato tubers, we reasoned that *Pcb*1692 T6SS could play a role in competition *in planta.* To determine the contribution of the *Pcb*1692 T6SS in bacterial competition, *in planta*, standardized cultures of *Pcb*1692, *Pcb*1692ΔT6, or *Pcb*1692ΔT6-pT6 were independently co-inoculated with targeted bacteria (*D. chrysanthemi, D. dadantii, Pcc*, or *Pcb*G4P5) into surface sterilized potato tubers, and CFU/ml of targeted bacteria enumerated 3 days post-inoculation. The results show no reduction in the CFU/ml of *Pcb*G4P5 when co-inoculated in potato tubers with either wild-type *Pcb*1692, *Pcb*1692ΔT6, or *Pcb*1692ΔT6-pT6 suggesting that T6SS-related effectors secreted by *Pcb*1692 cannot inhibit growth of this particular strain (Figure 8). However, a statistically significant (*p* < 0.05) 2–3 log reduction in the CFU/ml was observed when *D. dadantii*, *Pcc*, or *D. chrysanthemi* were co-inoculated with the wild-type *Pcb*1692 and *Pcb*1692ΔT6-pT6, this inhibition was completely lost when targeted strains were co-inoculated with *Pcb*1692ΔT6 (Figure 8). These results demonstrate the involvement of the *Pcb*1692 T6SS in interbacterial competition in potato tubers.

We earlier demonstrated that although the *in vitro* production of carbapenem by *Pcb*1692 plays a role in both intra and interspecies competition, it does not inhibit growth of *D. chrysanthemi*. The *D. chrysanthemi* strain used in this study was found to be resistant to ampicillin, suggesting that this strain might produce a β-lactamase which degrades carbapenem. Conversely, we found that the T6SS of *Pcb*1692 inhibits growth of *D. chrysanthemi in planta* but not *in vitro*. These findings together with our *in vitro* competition assays suggest that depending on the ecological niche, *Pcb*1692 can use either the T6SS or carbapenem to inhibit growth of several bacteria species. This is further supported by the fact that the *Pcb*1692 *car* gene cluster, unlike the T6SS, is not differentially expressed *in planta* and may thus be recruited by *Pcb*1692 to eliminate competitors while residing on the rhizosphere or plant surfaces (Bellieny-Rabelo et al., 2019b). However, following ingress into plant tissue *Pcb*1692 may recruit mainly the T6SS for bacterial competition.

### *Pcb*1692 Produces Bacteriocin(S) Following Mitomycin C Induction

**FIGURE 9:**
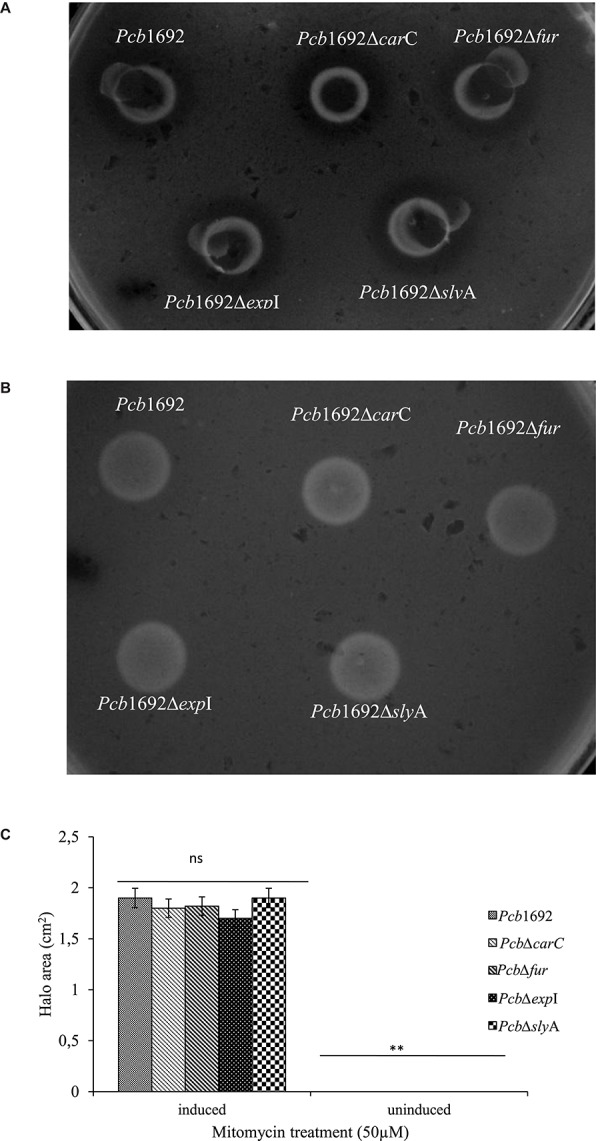
Bacteriocin production by strains of *Pectobacterium carotovorum* subsp. *brasiliense* 1692. *Pcb*1692 and its isogenic mutant strains were spotted on LB and grown overnight. **(A)** Bacteriocin production was induced with mitomycin C **(B)** control plate not induced. *Dickeya dadantii* was used as the indicator strain. Clear zones are indicative of bacteriocin production by *Pcb*1692. **(C)** Halo area was measured and ImageJ and data presented as the mean, error bars represent one standard deviation and asterisk represent statistical significant difference (*p* < 0.05) as determined by the two-tailed Student’s *t*-test. Experiments were performed in triplicates and repeated three independent times. Ns, not statistically significant.

**FIGURE 10:**
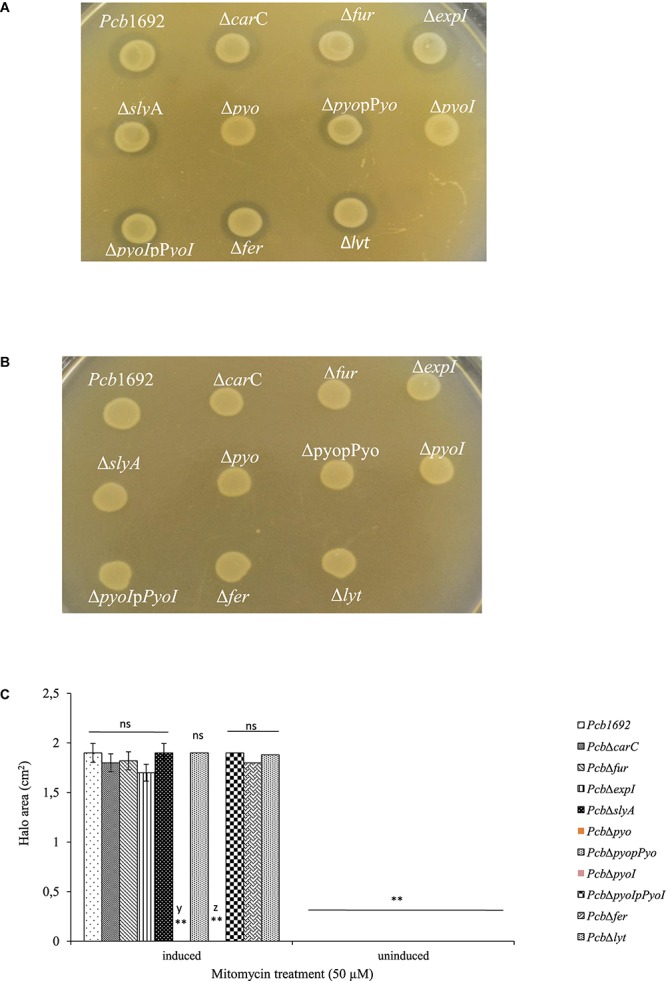
Production and detection of the S-type pyocin of *Pectobacterium carotovorum* subsp. *brasiliense* 1692. *Pcb*1692 and its isogenic mutant strains including *Pcb*1692Δ*pyo*, *Pcb*1692Δ*pyoI* and their respective complemented strains were spotted on LB and grown overnight. Bacteriocin production was induced with mitomycin C for 5 h. Induced cells were killed with chloroform vapor and overlaid with **(A)** a lawn of either *Pcb*1692Δ*pyoI* or **(B)** *Pcb*1692Δ*pyoI*p*pyoI*. Clear zones are indicative of bacteriocin production. **(C)** Halo area was measured and ImageJ and data presented as the mean, error bars represent one standard deviation and asterisk represent statistical significant difference (*p* < 0.01) as determined by the two-tailed Student’s *t*-test. Experiments were performed in triplicates and repeated three independent times. Ns, not statistically significant; y, *Pcb*1692Δ*pyo* and z, *Pcb*1692Δ*pyoI.*

Despite the fact that the genome sequence of *Pcb*1692 has genes encoding pyocin and carotovoricin, no role was determined for these bacteriocins in the assays we conducted thus far. Bacteriocin production is usually induced in response to DNA damaging agents which trigger SOS response (Chuang et al., 2007; Chan et al., 2011). Thus, we investigated the ability of *Pcb*1692 to produce these bacteriocins following induction with either mitomycin C or UV irradiation, using the spot-on-lawn overlay method (see section “Materials and Methods”). The results showed that wild-type *Pcb*1692, *Pcb*1692Δ*car*C, *Pcb*1692Δ*fur*, *Pcb*1692Δ*sly*A, and *Pcb*1692Δ*exp*I all produced bacteriocins following mitomycin C treatment and no bacteriocins were produced in control experiments (Figure 9). Considering that the above mentioned *Pcb*1692 mutant strains do not produce carbapenem, this indicated that the clear zones around the spotted bacteria may either represent *Pcb*1692 S-type pyocin, carotovoricin, a combination of both bacteriocins or a yet to be identified bacteriocin. In order to determine whether these clear zones were due to secretion of the putative S-type pyocin by *Pcb*1692, we generated a *Pcb*1692Δ*pyo*I double mutant strain lacking both the pyocin and putative immunity genes but with intact genes associated with carbapenem and carotovoricin production. Interestingly, when *Pcb*1692Δ*pyo*I was overlaid on mitomycin C-induced *Pcb*1692 strains, we observed clear zones of inhibition around wild-type *Pcb*1692 and all mutant strains used in this study except *Pcb*1692Δ*pyo*, which lacks the S-type pyocin but has the immunity gene (Figure 10). In addition, the complemented pyocin mutant strain *Pcb*1692Δ*pyo*p*pyo* was restored in its ability to inhibit growth of *Pcb*1692Δ*pyo*I similar to wild-type *Pcb*1692 while no *Pcb*1692 strain could kill the complemented *pyo*I mutant *Pcb*1692Δ*pyo*Ip*pyo*I (Figure 10). Together these findings demonstrate that following mitomycin C induction, *Pcb*1692 secretes the S-type pyocin, which inhibits growth of bacteria lacking the immunity factor. To evaluate the host range of killing by the *Pcb*1692 S-type pyocin, the overlay assay was repeated with different bacteria and the results show that bacteriocins produced by *Pcb*1692 can inhibit growth of *P. atrosepticum*, *Pcc, Pco*, *D. dadantii, S. typhimurium*, and *E. coli* but not the type strains of *S. marcescens* and *D. chrysanthemi* including some environmental strains of *Pcc* isolated from South Africa (Supplementary Table S4). The results also showed that the *Pcb*1692 carotovoricin isogenic mutant strains, *Pcb*1692Δ*fer* and *Pcb*1692Δ*lyt* produced bacteriocins similar to the wild-type. This could be because the *Pcb*1692 carotovoricin mutant strains still produced S-type pyocin or because the ferredoxin gene located within the *ctv* gene cluster of *Pcb*1692 may not be essential for carotovoricin production given that it is not conserved in the genome sequence of several SREs that have this gene cluster (Bellieny-Rabelo et al., 2019b). Similarly, the carotovoricin lytic cassette has three lytic genes associated with cell lysis and release of carotovoricin, thus presenting the possibility of functional complementation following deletion of any one the lytic genes, in our case, *Pcb*1692 Δ*lyt*.

### *Pcb*1692 Produces Bacteriocins but Not Carbapenem Under Anaerobic Conditions

**FIGURE 11:**
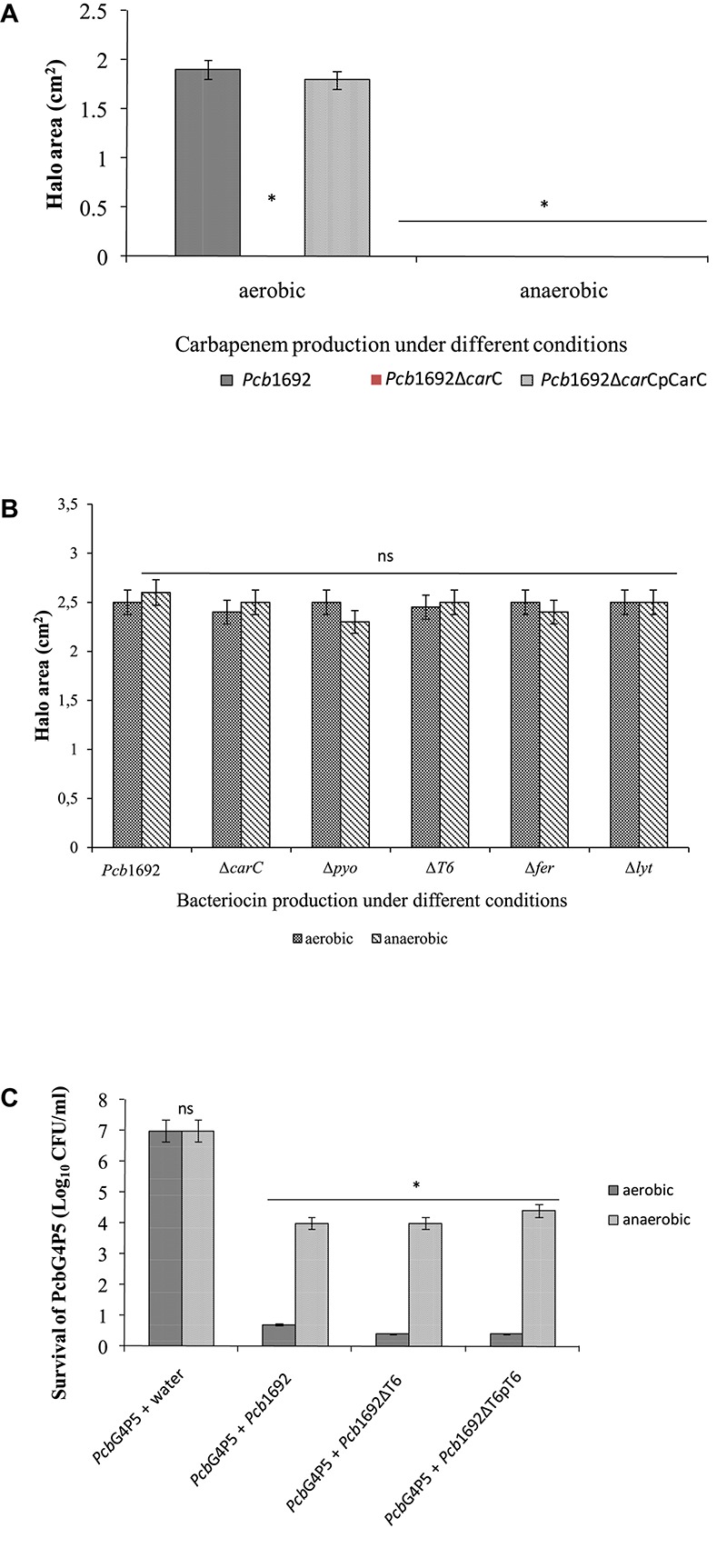
Effect of oxygen in competition, antibiotic and bacteriocin production by *Pectobacterium carotovorum* subsp. *brasiliense* 1692. **(A)** Carbapenem production by *Pcb*1692 strains spotted on a lawn of *Pcb*G4P5 and incubated aerobically and anaerobically. **(B)** Bacteriocin production by *Pcb*1692 strains induced with mitomycin C and overlaid with *Bacillus cereus.* **(C)** Interbacterial competition assays between *Pcb*1692 and the targeted strain *Pcb*G4P5, data is presented as CFU/ml of recovered targeted bacteria following 24 h incubation aerobically and anaerobically. Halo area was measured using ImageJ and data presented as the mean, error bars represent one standard deviation and asterisk represent statistical significant difference (*p* < 0.05) as determined by the two-tailed Student’s *t*-test. Experiments were performed in triplicates and repeated three independent times.

Previous studies have shown that oxygen is required for carbapenem production (Axelrood et al., 1988; Coulthurst et al., 2005). Therefore, to better understand the role played by oxygen in bacterial competition and the biological significance of carbapenem and bacteriocin production in the context of potato tubers, we assayed the ability of *Pcb*1692 to produce these antimicrobial compounds under aerobic or anaerobic conditions. The results demonstrated that *Pcb*1692 produces carbapenem under aerobic but not anaerobic conditions (Figure 11A). In addition, following mitomycin C treatment *Pcb*1692 and its isogenic mutant strains *Pcb*1692Δ*car*C, *Pcb*1692ΔT6, *Pcb*1692Δ*pyo*, *Pcb*1692Δ*fer*, and *Pcb*1692Δ*lyt* all produced bacteriocins when overlaid with either *Pcc*, *Pco*, *P. atrosepticum*, *D. dadantii* or *Bacillus cereus* under both aerobic and anaerobic conditions (Figure 11B and Supplementary Table S4). These findings clearly demonstrate that although bacteriocins are generally considered to have a narrow host range of killing (Riley and Gordon, 1999), bacteriocins produced by *Pcb*1692 may have a wide host range of killing which includes the *Firmicutes* and *B. cereus.* We further investigated the role played by the *Pcb*1692 T6SS in competition under anaerobic conditions, which mimics one of the conditions *in planta* where there is limited oxygen. Under aerobic conditions, no targeted bacteria were recovered when co-cultured with either *Pcb*1692 wild-type, mutant or complemented mutant strains. This could be ascribed to previously demonstrated production of *Pcb*1692 carbapenem under aerobic conditions (Figure 11A). *Pcb*1692 killing ability on target bacteria under anaerobic conditions was only slightly reversed in the *Pcb*1692ΔT6 mutant strain. Thus, we could conclude that the presence or absence of oxygen did not affect T6SS dependent *in vitro* bacterial competition (Figure 11C). Together these findings suggest that *Pcb*1692 might secrete bacteriocins but not carbapenem following ingress into potato tubers, where conditions are mostly anaerobic. Furthermore, the presence or absence of oxygen did not affect the magnitude of growth inhibition of targeted bacteria by *Pcb*1692ΔT6 relative to wild-type. This therefore supports our previous findings that the T6SS of *Pcb*1692 may only be functionally active *in planta*.

## Discussion

### Inducing Conditions Associated With Antimicrobial Production by *Pcb*1692

Bacteria exist as complex multispecies communities in any given niche (Kõiv et al., 2015; García-Bayona and Comstock, 2018). The interactions within these communities can be characterized by cooperation between species or more commonly, by competition (Stubbendieck and Straight, 2016; Chassaing and Cascales, 2018). Interference competition is mediated by production of an arsenal of antimicrobial weapons. In this study, we set out to explore microbe–microbe interactions that shape population structures of soft rot *Enterobacteriaceae* with a specific focus on interference competition. We used *Pcb*1692, an emerging soft rot pathogen of potatoes, as a model to help us understand factors shaping dynamics of microbial communities which can be found in decaying potato tubers. We were intrigued by the vast number of different antibacterial compounds encoded in the genome of *Pcb*1692 and other SREs, some of which were upregulated during *Pcb*1692 infection of potato tubers (Bellieny-Rabelo et al., 2019b). We hypothesized that these different systems likely target different types of bacteria and are likely deployed to enable *Pcb*1692 to colonize different environments. To test this hypothesis, we first performed a 16S metagenomics analysis of healthy and diseased potato tubers in order to determine the bacteria genus and strains (previously isolated from potato tubers in other studies) that can be used in competition against *Pcb*1692. The selected taxa used in this study such as *Serratia*, *Escherichia*, *Salmonella*, *Bacillus*, *Pseudomonas*, *Enterobacter*, *Pectobacterium*, and *Dickeya* represent bacteria that *Pcb* will likely encounter either in potato tubers, rhizosphere, or phyllosphere. Thereafter, we used a combination of *in silico* analyses, systematic mutagenesis of different antimicrobial systems and *in vitro* as well as *in planta* competition assays. To this end, we examined the role of different antimicrobial compounds produced by *Pcb*1692, including the T6SS, carbapenem, carotovoricin and bacteriocins, in bacteria competition. Our results identified some of the conditions associated with production of antimicrobial compounds by *Pcb*1692 such as (1) constitutive production of carbapenem *in vitro* under aerobic conditions, (2) iron-mediated secretion of carbapenem, under nutrient-limiting conditions, (3) SOS-mediated bacteriocin production, and (4) the role T6SS of *Pcb*1692 in bacteria competition when inoculated in potato tubers. This study sets an important milestone in interdisciplinary studies addressing the battery of compounds involved in both inter- and intra-specific bacterial competition.

The aforementioned inducing conditions mimic conditions likely encountered by*Pcb*1692 whether residing outside (both in the rhizo- and phyllo-sphere) or when colonizing intercellular spaces within potato tubers. For example, in the phyllosphere, availability of oxygen likely allows *Pcb* to produce carbapenem which inhibits growth of several *Proteobacteria* within the families *Enterobacteria* and *Pseudomonadaceae*, including *E. coli*, *Salmonella typhimurium*, *P. aeruginosa* and *Pcb* strains isolated from different diseased plant materials. While some of these bacteria represent enteric pathogens, members of the genera *Salmonella*, *Pseudomonas*, and *Escherichia* have previously been isolated from the rhizosphere and endosphere of several different plants including potato tubers, suggesting that *Pcb* and other SREs will compete with these bacteria (Hollis, 1951; Bulgarelli et al., 2012; Gupta et al., 2014; Schreiter et al., 2014; Donn et al., 2015; Hong et al., 2015; Kõiv et al., 2015). Similarly, the possibility of *Pcb* to encounter SOS-inducing conditions in the phyllosphere such as UV irradiation, nutrient limitation and production of antimicrobial compounds by other bacteria suggest that *Pcb* can potentially produce bacteriocins while residing in this niche. Once in the rhizosphere, *Pcb* encounters conditions of limited oxygen and SOS inducing conditions such as antimicrobial compounds produced by other bacteria (Erill et al., 2007; Goyal and Mattoo, 2014; Fang et al., 2017; Thorgersen et al., 2017; Cooper et al., 2018; García-Bayona and Comstock, 2018; Leaden et al., 2018), suggesting that *Pcb*1692 may be able to produce bacteriocins but not carbapenem while residing in the rhizosphere, however, this needs to experimentally verified. Finally, once inside potato tubers *Pcb* encounters several stresses which can trigger bacteria SOS such as nutrient starvation, iron deficiency, chromate stress, osmotic stress, oxidative and acidic stress, and β-lactam antibiotics produced by either bacteria endophytes or the host plant, leading to phage induction and bacteriocin production (Erill et al., 2007; Goyal and Mattoo, 2014; Fang et al., 2017; Thorgersen et al., 2017; Cooper et al., 2018; García-Bayona and Comstock, 2018; Leaden et al., 2018).

### Role of Carbapenem and Bacteriocin in Bacteria Competition, Resistance Mechanisms

Our results show that the different antimicrobials produced by *Pcb*1692 target different genera. For example, *Pcb*1692 deploys carbapenem and bacteriocins to inhibit growth of all the aforementioned targeted bacteria, except *S. marcescens*, *D. chrysanthemi*, and *P. wasabiae*. Our *in silico* analysis showed that some of the SREs analyzed in this study such as *D. dadantii*, *Pco*, and *P. atrosepticum* do not have genes associated with carbapenem production or resistance, which could account for the observed 5-log reduction in the CFU/ml of these strains when co-cultured with *Pcb*1692 (Figure 2). Similarly, the genome sequences of several closely related strains of *Pcb* do not have homologs of *car* genes which could in part explain the growth inhibition observed when *Pcb*1692 was co-cultured with *Pcb* (XT3, XT10, G4P5, and G4P7). In this regard, PCR amplification failed to amplify the *car*F and *car*G genes in *Pcb* (XT3, XT10, G4P5, and G4P7), however, because of the possibility of sequence variation and genetic heterogeneity of *Pcb* strains and SREs in general (Lee et al., 2014; Adeolu et al., 2016), one cannot rule out the presence or absence of a gene in a given bacterial genome solely based of PCR. The inability of the *Pcb*1692 carbapenem to inhibit growth of *D. chrysanthemi* and *P. wasabiae* and *S. marcescens* may be explained as follows: (1) The *in silico* analysis showed that although the genome sequences of *D. chrysanthemi* and *P. wasabiae* do not contain gene homologs associated with carbapenem biosynthesis genes they do contain homologs of *car*F and *car*G associated with carbapenem resistance, (2) although *in silico* analysis data we presented did not include analysis of the genome sequence of *S. marcescens* ATCC 13880^*T*^ (Daligault et al., 2014), our analysis indicated that the genome sequence of this strain has homologs of *car*A (*GSMA_00116*) and *car*B (*GSMA_00117*) but missing the remaining *car* genes. Additionally, carbapenem resistance has been observed in several *Proteobacteria* including *Klebsiella* and *Serratia* and this resistance has been associated with either production of β-lactamases, the constitutive expression of efflux pumps, reduced membrane permeability, or modification of penicillin-binding protein targets (Yang et al., 2012; Lutgring and Limbago, 2016). The ability of any bacteria to deploy either a single or a combination of the aforementioned carbapenem resistance mechanisms may in part explain the differences observed in the magnitude of inhibition shown in Figure 2. Similarly, following induction with mitomycin C, *Pcb*1692 produces bacteriocins which inhibit growth of several *Proteobacteria* and the *Firmicutes* and *B. cereus*. Our *in silico* analysis showed that homologs of the *Pcb*1692 pyocin and immunity factor are not conserved in genome sequences of most SREs analyzed including some closely related strains of *Pcb*. While this finding could in part be explained the observed killing (halo formation) of these strain, it does not explain resistance of the *S. marcescens*, *P. wasabiae*, and *D. chrysanthemi* to these bacteriocins. Like carbapenem resistance, it is possible that these bacteria may have developed different mechanisms associated with bacteriocin resistance such as phage superinfection, modification of membrane, DNA or RNA target sites or rapid efflux or neutralization of bacteriocins (Jarvis, 1967; Ming and Daeschel, 1993; Mazzotta et al., 1997; Hyman and Abedon, 2010).

### The Role Played by the *Pcb*1692 T6SS in Bacteria Competition

Previous studies have shown that while the T6SS of some bacteria is constitutively expressed and actively secretes antimicrobial compounds under laboratory conditions, the same system needs to be induced or activated in other bacteria by different cues such as temperature, osmolarity, oxygen, pH, plant extracts, and several divalent metals including iron (Miyata et al., 2013). For example, some T6SS genes of *Pectobacterium atrosepticum* are upregulated in the presence of plant extracts while the T6SS of *Pcb*1692 and *Dickeya* is massively upregulated when inoculated into potato tubers (Mattinen et al., 2007; Bellieny-Rabelo et al., 2019b; Raoul des Essarts et al., 2019). However, for SREs and most other plant pathogens, the exact role this secretion system plays in bacterial competition remains largely unknown (Bernal et al., 2018). Our results demonstrate that the *Pcb*1692ΔT6SS was attenuated in its ability to inhibit *D. chrysanthemi*, *D. dadantii* and *Pcc*, relative to wild-type when co-inoculated into potato tubers. These findings clearly demonstrate that *Pcb*1692 uses the T6SS to inhibit growth of *D. chrysanthemi in planta* but not *in vitro.* Therefore, it is possible that this system is recruited to inhibit growth of some endophytes that likely share the ‘potato tuber niche’ with *Pcb*. On the other hand, the inability of the T6SS of *Pcb*1692 to inhibit growth of the closely related *Pcb*G5P7 may be due to the presence of overlapping T6-related toxin/immunity modules in their genome. This hypothesis is supported by the reported conservation of one of the T6-islands encoding several predicted toxin/antitoxin modules in different strains of *Pcb* (Bellieny-Rabelo et al., 2019b). Overall, our analysis suggests that once inside potato tubers, the T6SS of *Pcb*1692 is activated and alongside its arsenal of bacteriocins, inhibits growth of targeted bacteria.

### The Role Played by Iron, Oxygen, and Fur on Carbapenem Production

Iron is an essential element which acts as a cofactor of several enzymes, a constituent of heme and is required for biosynthesis of iron–sulfur clusters (Fe–S) found in several proteins (Andrews et al., 2003; Seo et al., 2014). Because of its importance, bacteria and their host plants are continuously engaged in an arms race for iron. During these interactions, the host plant limits iron availability to invading microbes by sequestering iron in proteins such as ferritin while the bacteria by producing siderophore, which are high-affinity iron uptake systems capable of chelating iron from the plant (Boughammoura et al., 2007; Skaar, 2010). Despite its involvement in several biological processes, both excess and limited iron is detrimental to bacteria. Furthermore, intracellular levels of iron are regulated by the ferric uptake regulator (Fur) (Chandrangsu et al., 2017). In the presence of iron, Fur binds to iron and the Fur^*Fe*2+^ complex represses expression of several genes including iron uptake gene while concomitantly activating expression of genes associated with iron efflux, virulence, stress response and several metabolic processes (Foster, 1991; Andrews et al., 2003; Delany et al., 2004; Hassan and Troxell, 2013; Seo et al., 2014; Porcheron and Dozois, 2015). Interestingly, our results showed that carbapenem production by *Pcb*1692 is dependent on iron and may be regulated directly or indirectly by Fur. This is based on the fact that *Pcb*1692Δ*fur* mutant strain lost the ability to produce carbapenem compared to the wild-type strain. Similarly, the results showed that wild-type *Pcb*1692 only produced carbapenem when M9 was supplemented with iron. Identification of putative Fur binding sites in *sly*A and *car*R promoter regions in *Pcb*1692, *Pcc*, *Pco*, and *Pca* further suggests that Fur might indirectly regulate expression of the carbapenem genes by binding to the promoter sequences of *sly*A and *car*R. In addition to iron and Fur, our results demonstrate that *Pcb*1692 produces detectable levels of carbapenem and bacteriocins when spotted on a lawn of targeted bacteria and incubated aerobically, however, when the above assay was repeated under anaerobic conditions, *Pcb*1692 lost the ability to produce carbapenem but not bacteriocins.

The requirement for iron and Fur in the biosynthesis of carbapenem is consistent with the finding that CarE is a putative ferredoxin with 2 Fe–S clusters while the carbapenem synthase CarC protein is an oxygenase which requires iron and oxygen and 2-oxoglutarate as co-factors (Hamed et al., 2013; Dunham et al., 2018). The requirement for oxygen by CarC is also supported by our findings which showed that that *Pcb*1692 does not produce carbapenem under anaerobic conditions. Therefore, the effect of iron and oxygen may have a direct effect on functionality of CarC and CarE and hence carbapenem production in the apoplast where there is limited iron and oxygen. It is important to note that the ability of *Pcb* and SREs in general to produce carbapenem and bacteriocins *in planta* has not been determined in this study including previous studies partly because of the difficulty associated with identification and quantification of antibiotics and bacteriocins produced by bacteria *in planta*. Therefore, we can only cautiously speculate on the physiological roles these compounds play during *in planta* bacteria competition based on the assay conditions which mimic *in planta* conditions conducted in the current study.

## Conclusion

Our results demonstrate that *Pcb* deploys an arsenal of antimicrobial compounds targeted at different bacteria and recruited under different environmental conditions to give *Pcb*1692 a competitive advantage over different endophytes likely encountered during various stages of potato tuber invasion. This competitive advantage spans phyllosphere which is typically aerobic, rhizosphere and intercellular spaces inside potato tubers which are typically characterized by anaerobiosis and iron-limiting conditions. For example, the *Pcb*1692 T6SS is recruited *in planta* following perception of unknown plant signals and inhibits members of the *Enterobacteriaceae*. Furthermore, *Pcb*1692 produces carbapenem and bacteriocins, which inhibit several gram-negative and gram-positive bacteria including *B. cereus*. While *Pcb*1692 can produce bacteriocins aerobically and anaerobically, carbapenem production was found to be dependent on the availability of oxygen and iron. Finally, the results showed that carbapenem production by Pcb1692 is regulated by the concerted action of Fur, SlyA and ExpI.

## Data Availability Statement

The 16S metagenomics raw data has been deposited in the Sequence Read 567 Archive database on NCBI under the Accession No. PRJNA509544.

## Author Contributions

DS and LM conceived the study and designed the experiments. NS performed the metagenomic study. DB-R analyzed metagenomics data and performed the *in silico* analysis. DS, NN, and AG performed the experiments. DS, DB-R, and LM analyzed the data and provided technical and scientific discussion. DS wrote the manuscript. NN, AG, DB-R, and LM revised the manuscript. All authors read and approved the final manuscript.

## Conflict of Interest

The authors declare that the research was conducted in the absence of any commercial or financial relationships that could be construed as a potential conflict of interest.

## Footnotes

1 www.ncbi.nlm.nih.gov
