# Supplementary material for: The Impact of Type VI Secretion System, Bacteriocins and Antibiotics on Bacterial Competition of Pectobacterium carotovorum subsp. brasiliense and the Regulation of Carbapenem Biosynthesis by Iron and the Ferric-Uptake Regulator
Source: Front Microbiol. 2019 Oct 18;10:2379. doi: 10.3389/fmicb.2019.02379 (PMC6813493; doi:10.3389/fmicb.2019.02379)
Supplement: Supplementary file 2 [file Data_Sheet_2.docx]

**Supplementary Data Sheet S2. The effect of different concentrations of iron on the outcome of bacteria competition between *Pectobacterium carotovorum* subsp. *brasiliense* 1692 and targeted bacteria.** *Pcb*1692 and targeted bacteria were co-cultured in a 1:1 ratio on M9 minimal media supplement with different concentrations of iron. Survival of targeted bacteria was determined by 10 fold serial dilutions and the log_10_CFU/ml of surviving targeted bacteria determined 24hrs post incubation. Experiments were performed in triplicates and repeated three independent times. Bars represent mean values, error bars represent one standard deviation and P values for the differences in log_10_CFU/ml of recovered target bacteria have been provided as determined by the two-tailed Student’s *t*-test. *P*<0.05 was considered to be statistically significant. Pcc = *Pectobacterium carotovorum* subsp. *carotovorum*, Pco = *Pectobacterium carotovorum* subsp. *odoriferum*, Dada = *Dickeya dadantii*.
