# Supplementary material for: The Impact of Type VI Secretion System, Bacteriocins and Antibiotics on Bacterial Competition of Pectobacterium carotovorum subsp. brasiliense and the Regulation of Carbapenem Biosynthesis by Iron and the Ferric-Uptake Regulator
Source: Front Microbiol. 2019 Oct 18;10:2379. doi: 10.3389/fmicb.2019.02379 (PMC6813493; doi:10.3389/fmicb.2019.02379)
Supplement: Supplementary file 3 [file Data_Sheet_3.docx]

**A**


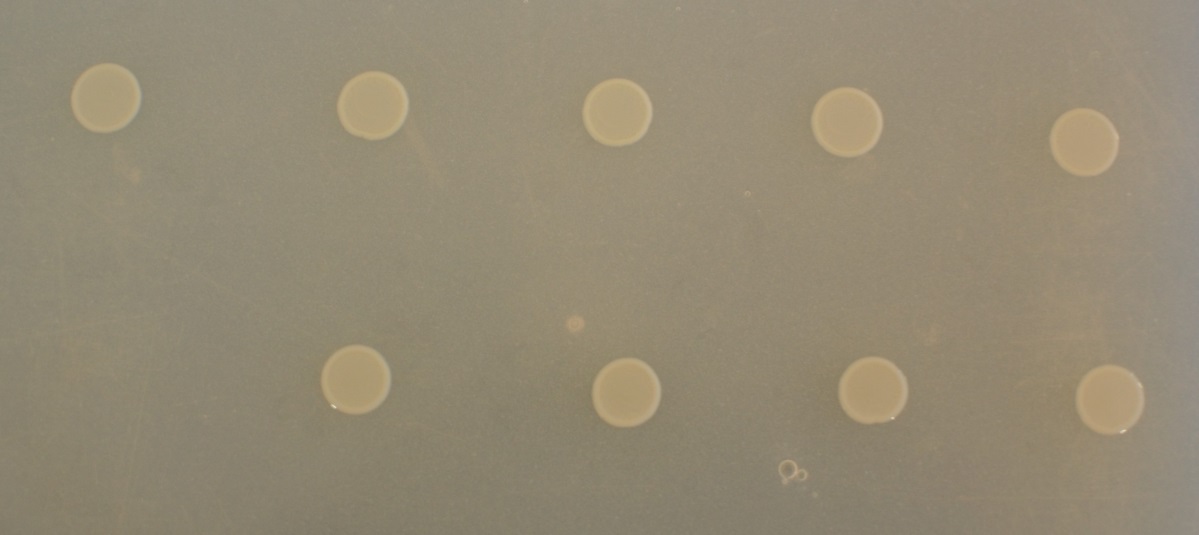


∆*exp*I

∆*sly*A

∆*car*C

∆*exp*Ip*Exp*I

∆*sly*Ap*Sly*A

∆*car*Cp*Car*C

∆*furpFur*

∆*fur*

*Pcb*1692

**B**


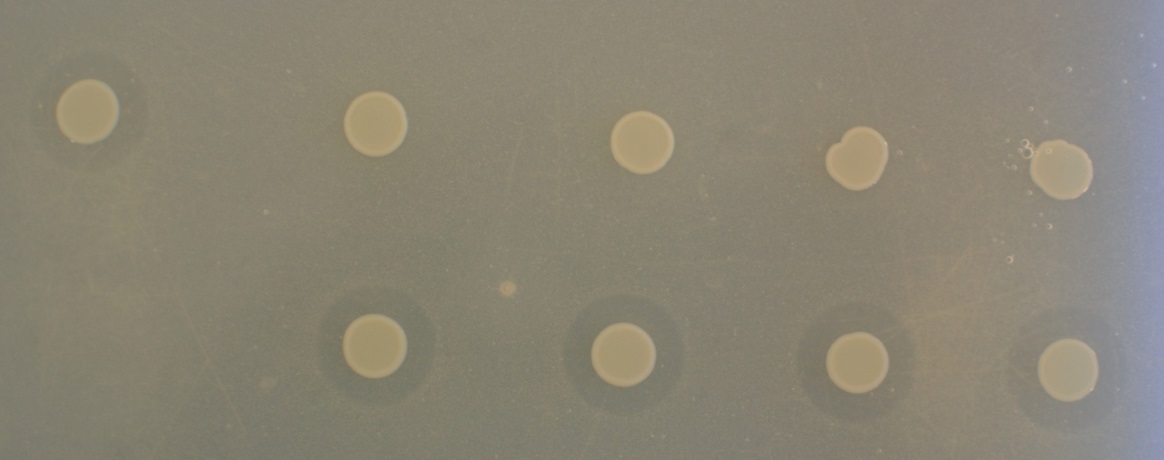


∆*exp*I

∆*exp*Ip*Exp*I

∆*fur*p*Fur*

∆*car*Cp*Car*C

∆*sly*Ap*Sly*A

∆*sly*A

∆*car*C

∆*fur*

*Pcb*1692

**C**

**Supplementary Data Sheet S3. The effect of iron on carbapenem production *Pectobacterium carotovorum* subsp. *brasiliense* 1692 and targeted bacteria.** M9 minimal media was supplemented with or without iron (10µM). *Pcb*1692 and its corresponding mutant and complement strains were spotted on a lawn of *Dickeya dadantii* and incubated overnight at 28^o^C A) No halos were produced by strains *Pcb*1692 in the absence of iron. B) Halos were observed in wildtype *Pcb*1692 and complement mutant strains which was indicative of carbapenem production. C) Halo area was measured using ImageJ and data presented as the mean, error bars represent one standard deviation and asterisk represent statistical significant difference (*p*<0.05) as determined by the two-tailed Student’s *t*-test. Experiments were performed in triplicates and repeated three independent times.
