# Supplementary material for: The Impact of Type VI Secretion System, Bacteriocins and Antibiotics on Bacterial Competition of Pectobacterium carotovorum subsp. brasiliense and the Regulation of Carbapenem Biosynthesis by Iron and the Ferric-Uptake Regulator
Source: Front Microbiol. 2019 Oct 18;10:2379. doi: 10.3389/fmicb.2019.02379 (PMC6813493; doi:10.3389/fmicb.2019.02379)
Supplement: Supplementary file 4 [file Table_1.doc]

**Supplementary Table S1. List of bacterial strains used in this study:** UK = United Kingdom, USA = United States of America, FABI = Forestry and Agricultural Biotechnology Institute, University of Pretoria. Bacterial strains whose genome sequence has been determined are indicated as “sequenced” in the Description column. Bacteria type strains and indicated by the superscript T (^T^) following the bacterial name. *P* = *Pectobacterium*, *Pcc* = *Pectobacterium carotovorum* subsp. *carotovorum.*

| **Bacterial strains** | **Description (Host and country of isolation)** | **Sources** |
| --- | --- | --- |
| **Proteobacteria** | | |
| *Pectobacterium carotovorum* subsp. *brasiliense* (*Pcb*) |  |  |
| *Pcb* 1692 | Potato, Brazil, sequenced | (Glasner et al., 2008; Charkowski, 2018) |
| *Pcb* G4P5 | Potato, South Africa | FABI |
| *Pcb* G4P7 | Potato, South Africa | FABI |
| *Pcb* XT3 | Potato, South Africa | FABI |
| *Pcb* XT10 | Potato, South Africa | FABI |
| *Pcb* 358 | Potato, South Africa | FABI |
| *Pcb* CC1 | Cucumber, South Africa | FABI |
| *Pcb* CC2 | Cucumber, South Africa | FABI |
| *Pcb* HPI01 | Cucumber, South Africa, sequenced | (Onkendi et al., 2016) |
| *Pcc*1 | Unknown, South Africa | FABI |
| *Pcc*2 | Unknown, South Africa | FABI |
| *Pcc*BR1 | Unknown, South Africa | FABI |
| *Pcc*BR3 | Unknown, South Africa | FABI |
| *Pcc*BR7 | Unknown, South Africa | FABI |
| *Pcc*RP6P2 | Unknown, South Africa | FABI |
| *Pcc*GP6P1 | Unknown, South Africa | FABI |
| *Pcc*YP10R2 | Unknown, South Africa | FABI |
| *Pcc*RP17P1 | Unknown, South Africa | FABI |
| *Pcc*RP17P2 | Unknown, South Africa | FABI |
| *P. atrosepticum* ATCC 33260^T^ | Potato, UK, sequenced | (Panda et al., 2015) |
| *P. carotovorum* ATCC 15713^T^ | Potato, sequenced | (Gardan et al., 2003) |
| *P. wasabiae* ATCC 43316^T^ | *Eutrema wasabi*, Japan, sequenced | (Goto and Matsumoto, 1987) |
| *P. cypripedii* PDDCC 1591^T^ | *Cypripedium sp.,*USA | (Hori, 1911) |
| ***Dickeya paradisiaca*** ATCC 33242^T^ | *Musa paradisiaca* var. *dominico*, Colombia,sequenced | (Hauben et al., 1998) |
| *P. betavasculorum* LMG 2398 | Potato, Romania | FABI |
| *P. carotovorum* subsp*. odoriferum* | Unknown, South Africa | FABI |
| *Salmonella typhimurium* | Unknown, South Africa | FABI |
| *Pseudomonas aeruginosa* | Unknown, South Africa | FABI |
| *Escherichia coli* | Unknown, South Africa | FABI |
| *Dickeya dadantii* LMG 25991^T^ | *Pelargonium capitatum,* Comoros*,* sequenced | (Samson et al., 2005) |
| *Dickeya chrysanthemi* ATCC 11663^T^ | *Chrysanthemum*, USA*,* sequenced | (Burkholder et al., 1953) |
| *Erwinia rhapontici* LMG 2645 | Pear, UK | FABI |
| *Serratia marcescens* subsp. *marcescens* ATCC 13880^T^ | Pond water, Czech Republic, sequenced | (Daligault et al., 2014) |
| *Pantoea ananatis LMG2665^T^* | Onion, Brazil, sequenced | (Serrano, 1928) |
| *Pantoea stewartii subsp. indologenes ATCC 51785*^T^ | Setaria italic, India, sequenced | (Popp et al., 2010) |
| *Enterobacter cowanii* | Eucalyptus grandis, Uruguay | FABI |
| *Serratia fiscaria* ATCC 33105^T^ | Fig | (Grimont et al., 1981) |
| **Firmicutes** |  |  |
| *Bacillus subtilis* | Unknown | FABI |
| *Bacillus cereus* | Unknown | FABI |
