# Supplementary material for: The Impact of Type VI Secretion System, Bacteriocins and Antibiotics on Bacterial Competition of Pectobacterium carotovorum subsp. brasiliense and the Regulation of Carbapenem Biosynthesis by Iron and the Ferric-Uptake Regulator
Source: Front Microbiol. 2019 Oct 18;10:2379. doi: 10.3389/fmicb.2019.02379 (PMC6813493; doi:10.3389/fmicb.2019.02379)
Supplement: Supplementary file 5 [file Table_2.doc]

**Supplementary Table S2. List of primers used in the *Pcb*1692 mutagenesis and complementation:** The primer combinations ccarFF and ccarFR amplify the *Pcb*1692 *car*C gene, while the primers designated and icarFF/ icarGR amplify the carbapenem immunity genes *car*FG. The underlined primer sequence contains the *SpeI* restriction enzyme site for cloning into plasmid vectors. Amp^r^ = ampicillin resistance gene.

| **PRIMERS** | **SEQUENCE (5’-3’)** | **Source** |
| --- | --- | --- |
| **16S rRNA metagenomics primers** | | |
| 419F | ACTCCTACGGGAGGCAGCAG | (1) |
| 806R | GGACTACHVGGGTWTCTAAT | (1) |
| **T6SS mutagenesis and complementation primers (***PCBA_RS11175::PCBA_RS11170*) tail sheath proteins TssBC | | |
| tsscF | TTCAGCACGCTGGCTTCCTG | This study |
| tsscRK | CGAAGCAGCTCCAGCCTACACAGAACTTTGAGCTGTCGCTGGTAG | This study |
| kanF | CTACCAGCGACAGCTCAAAGTTCTGTGTAGGCTGGAGCTGCTTCG | This study |
| tsscFK | CTAAGGAGGATATTCATATGATGTGTCTATACCAACGTAAG | This study |
| kanR | CTTACGTTGGTATAGACACATCATATGAATATCCTCCTTAG | This study |
| tsscR | TGAACTTCTCTAGGCATTCTGC | This study |
| testR | TGTCCTTCACTAATTAGTTGG | This study |
| testF | ATGCTGGAAGAGATTGATTGC | This study |
| compF | GCATAAGTCTTATTCCTTATCC | This study |
| compR | TCAGCGTGGATTGTAGTGTAATC | This study |
| **Carotovoricin mutagenesis and complementation primers (Ferrodoxin & Lytic transglycolysase)** | | |
| 1. Ferrodoxin (*PCBA_RS08670*) | | |
| TferF | CAGGGCATCAGCACCATTTTC | This study |
| ferF1 | CCTGGCCAACCGTGACGCACATG | This study |
| ferR1 | CGAAGCAGCTCCAGCCTACACAAAATACTTTTGCTGACATAGTC | This study |
| kanFfer | GACTATGTCAGCAAAAGTATTTTGTGTAGGCTGGAGCTGCTTCG | This study |
| KanRfer | GATAATTAATGATGCAGCTCATCTTCCATATGAATATCCTCCTTAGTTCC | This study |
| ferF2 | GGAACTAAGGAGGATATTCATATGGAAGATGAGCTGCATCATTAATTATC | This study |
| ferR2 | GAAATGGACTCCACGCGTTGAC | This study |
| FERRR2 | ACTCGGCTGAAAGCGAAACCAC | This study |
| TferR | TCGGGATGTCACCTGTGTGC | This study |
| **2) Lytic transglycosylase (***PCBA_RS08630*) | | |
| TlytF | GGTTCTGTCTCCGCATCTATG | This study |
| TlytR | GTCGGATACGCTGCGTATCAGC | This study |
| lytF | GCAAAGTCAGGCTGAGCTGGAC | This study |
| lytR | ATCCAGCTTGGCTTCGATGAC | This study |
| lytFCHL | GGATATTAATACTGAAACTGGGAATAGTCTGTTTCTCAATAGC | This study |
| CHLRlyt | GCTATTGAGAAACAGACTATTCCCAGTTTCAGTATTAATATCC | This study |
| lytRCHL | CCAATAGGATATCGGCATTTTCTTTTGCGTTT CATATCACAACCCCATCGCTACGC | This study |
| CHLFlyt | GCGTAGCGATGGGGTTGTGATATGAAACGCAAAAGAAAATGCCGATATCCTATTGG | This study |
| **Carbapenem mutagenesis primers** (*PCBA_RS04375*[*carC*]) | | |
| TcarF | TCGATTGGGACGGAACTCAGC | This study |
| carF | TGTACCGGACGCGTTGGACAG | This study |
| carR | CAACAGCGTTCTCTTCAATCC | This study |
| TcarR | TGAACTGAGCACGCTTTCATC | This study |
| carKR | CGAAGCAGCTCCAGCCTACACACTTCACTATTTCGCTCATTTTCTTCC | This study |
| KcarF | GGAAGAAAATGAGCGAAATAGTGAAGTGTGTAGGCTGGAGCTGCTTCG | This study |
| carKF | GGAACTAAGGAGGATATTCATATGCAAACCGCCGATATCTAACTCTC | This study |
| KcarR | GAGAGTTAGATATCGGCGGTTTGCATATGAATATCCTCCTTAGTTCC | This study |
| **Pyocin Mutagenesis Primers** (1-pyocin*PCBA_RS02805* and 2-pyocin and immunity (*PCBA_RS02805:: PCBA_RS02810*) | | |
| pyocinF | ACGGAATAATCGGATATTGAC | This study |
| pyocinKR | GAACTTCGAAGCAGCTCCAGCCTACACAAGCATATAAACGGTACTTAATC | This study |
| pyocinKF | GGAACTAAGGAGGATATTCATATGGGTGTTAAGATGAGTAGTAAACTTATTG | This study |
| KpyocinF | GATTAAGTACCGTTTATATGCTTGTGTAGGCTGGAGCTGCTTCGAAGTTC | This study |
| KpysosinR | CAATAAGTTTACTACTCATCTTAACACCCATATGAATATCCTCCTTAGTTCC | This study |
| pyocinR | TTACCAATCAGGTAGTTTGTC | This study |
| TpyocinR | TCAATAGATGGTGATAGTCAC | This study |
| TpyocinF | AGCATGTCTTTAATCACCTGAC | This study |
| immF | GGAACTAAGGAGGATATTCATATGTATCTCTTGCGTGTAAGAAAGATACGC | This study |
| KimmR | GCGTATCTTTCTTACACGCAAGAGATACATATGAATATCCTCCTTAGTTCC | This study |
| compF | ATCTGTGCTCGTTGCAAAAGG | This study |
| compR | GTTTACTACTCATCTTAACACC | This study |
| compimmR | AGCGCGTATCTTTCTTACACG | This study |
| **Carbapenem complementation primers** | | |
| CcarCF | AAAAACTAGTGGATAAGGAAGAAAATGAGCG | This study |
| CcarCR | AAAAACTAGTAGATCCATATCAATCCGGCAC | This study |
| IcarFF | AAAAACTAGTGTGAATGCTGTTCCTGACGAG | This study |
| IcarGR | AAAAACTAGTTCATTTTGCCAGCATCGCCTC | This study |
| **Tetracyline primers** | | |
| TETF | GATGTTGTCTACATGGCTCTG | This study |
| TETR | TGGGCGGTGACGTCGTTCGAG | This study |
|  | **FUR PROMOTER PRIMERS** |  |
| FurF | TTGGCTAAGCCATTTCGGCTC | This study |
| FurR | AAAAACTAGTGCGGGTTTAATCCTGTTGCTTAC | This study |
|  | KANAMYCIN TEST PRIMERS |  |
| Km1 | CAGTCATAGCCGAATAGCCT | (2) |
| Km2 | CGGTGCCCTGAATGAACTGC | (2) |
| **Inverse PCR primers To Remove Amp^r^ From CloneJet1.2** | | |
| IRF | GTTGAATACTCATACTCTTC | This study |
| IRR | AGCATTGGTAACTGTCAGACC | This study |

1. Fadrosh DW, Ma B, Gajer P, Sengamalay N, Ott S, Brotman RM, Ravel J. 2014. An improved dual-indexing approach for multiplexed 16S rRNA gene sequencing on the Illumina MiSeq platform. Microbiome 2:6.

2. Datsenko KA, Wanner BL. 2000. One-step inactivation of chromosomal genes in Escherichia coli K-12 using PCR products. Proceedings of the National Academy of Sciences 97:6640-6645.
