# Supplementary material for: The Impact of Type VI Secretion System, Bacteriocins and Antibiotics on Bacterial Competition of Pectobacterium carotovorum subsp. brasiliense and the Regulation of Carbapenem Biosynthesis by Iron and the Ferric-Uptake Regulator
Source: Front Microbiol. 2019 Oct 18;10:2379. doi: 10.3389/fmicb.2019.02379 (PMC6813493; doi:10.3389/fmicb.2019.02379)
Supplement: Supplementary file 6 [file Table_3.doc]

**Supplementary Table S3. List of *Pcb*1692 mutant strains and complements generated in this study:** Kan*^r^* = kanamycin resistance, Tet^r^ = tetracycline resistance, Cm^r^ = chloramphenicol resistance.

| **Bacterial strains** | **Description** | **Sources** |
| --- | --- | --- |
| *Pectobacterium carotovorum* subsp. *brasiliense* 1692 (*Pcb*1692) | Isolated from potato in Brazil, sequenced strain | (Duarte et al., 2004; Glasner et al., 2008) |
|  |  |  |
| *Pcb*1692ΔT6SS | *Pcb*1692 double mutant with a deletion of both the *tss*A and *tss*B genes located within the major *Pcb*1692 T6SS gene cluster*,* Kan^r^ | This study |
| *Pcb*1692Δ*lyt* | *Pcb*1692 with a deletion in the gene encoding the lytic transglycosylase gene located within the carotovorin gene cluster, Cm^r^ | This study |
| *Pcb*1692Δ*fer* | *Pcb*1692 with a deletion in the gene encoding ferredoxin located within the carotovorin gene cluster, Kan^r^ | This study |
| *Pcb*1692Δ*car* | *Pcb*1692 with a deletion in the gene encoding ferredoxin located within the carbapenem gene cluster, Kan^r^ | This study |
| *Pcb*1692Δ*pyo* | *Pcb*1692 with a deletion in the gene encoding a putative S-type pyocin, Kan^r^ | This study |
| *Pcb*1692Δ*pyo*I | *Pcb*1692 double mutant with a deletion in genes encoding pyocin and the downstream immunity gene, Kan^r^ | This study |
| *Pcb*1692Δ*fur* | *Pcb*1692 with a deletion in the gene encoding the transcriptional regulator Fur | (Tanui et al., 2017) |
| *Pcb*1692Δ*exp*I | *Pcb*1692 with a deletion in the gene encoding the AHL synthase protein, ExpI | (Moleleki et al., 2017) |
| *Pcb*1692Δ*sly*A | *Pcb*1692 with a deletion in the gene encoding the stress response regulator SlyA | (Bellieny-Rabelo et al., 2019) |
| *Pcb*1692Δ*sly*Ap*sly*A | *Pcb*1692Δ*sly*A expressing the *sly*A gene from pJET3plasmid;Kan^r^, Tet^r^ | This study |
| *Pcb*1692ΔT6pT6 | *Pcb*1692Δ(*tss*B::*tss*C) expressing *tss*B::C genes from pJET3 plasmid;Kan^r^, Tet^r^ | This study |
| *Pcb*1692Δ*pyo*p*pyo* | *Pcb*1692Δ*pyocin* expressing thepyocin gene from pJET3 plasmid;Kan^r^, Tet^r^ | This study |
| *Pcb*1692Δ*pyo*Ip*pyo*I | *Pcb*1692Δ*pyoI* expressing pyocin and immunity genes from pJET3 plasmid;Kan^r^, Tet^r^ | This study |
| *Pcb*1692Δ*fur*p*fur* | *Pcb*1692Δ*fur* expressing the *fur* gene from pJET3 plasmid;Kan^r^, Tet^r^ | This study |
| *Pcb*1692Δ*exp*Ip*exp*I | *Pcb*1692Δ*exp*Iexpressing the *exp*I gene from pJET3 plasmid;Kan^r^, Tet^r^ | This study |
| *Pcb1692*Δ*car*Cp*car*C | *Pcb*1692Δ*car*Cexpressing the *car*C gene from pJET4 plasmid;Kan^r^, Tet^r^ | This study |
| **Plasmids** | | |
| pKD4 | Plasmid containing a Kan^r^ cassette | (Datsenko and Wanner, 2000) |
| pKD20 | Plasmid expressing the lambda red genes | (Datsenko and Wanner, 2000) |
| pREDTER | Plasmid the lambda red genes, Cm^r^ | (Katashkina et al., 2009) |
| pMP7605 | pBRR replicon, broad host range vector, Gm^r^ | (Lagendijk et al., 2010) |
| pME6031 | Broad host range vector, Tet^r^ | (Heeb et al., 2000) |
| pJET1.2/blunt | Bacterial cloning vector containing | ThermoFisher Scientific |
| pJET3 | Modified pJET1.2/blunt in which the entire amp^r^ sequence was deleted and replaced with Tet^r^, Tet^r^ | This study |
| pJET4 | Modified pJET3 expressing the *Pcb*1692 Fur promoter sequence, Tet^r^ | This study |
| pJET4-*car*C | Bacterial expression vector expressing the *car*C gene from the Fur promoter, Tet^r^ |  |
| pJET4-*car*FG | Bacterial expression vector expressing the *car*C and *car*F genes from the Fur promoter, Tet^r^ | This study |
|  |  |  |

Bellieny-Rabelo, D., Nkomo, N.P., Shyntum, D.Y., and Moleleki, L.N. (2019). Horizontally acquired quorum sensing regulators recruited by the PhoP regulatory network expand host-adaptation repertoire in the phytopathogen Pectobacterium carotovorum. *bioRxiv***,** 776476.

Datsenko, K.A., and Wanner, B.L. (2000). One-step inactivation of chromosomal genes in Escherichia coli K-12 using PCR products. *Proceedings of the National Academy of Sciences* 97(12)**,** 6640-6645.

Duarte, V., de Boer, S.H., Ward, L.J., and de Oliveira, A.M. (2004). Characterization of atypical Erwinia carotovora strains causing blackleg of potato in Brazil. *J Appl Microbiol* 96(3)**,** 535-545.

Glasner, J., Marquez-Villavicencio, M., Kim, H.-S., Jahn, C., Ma, B., Biehl, B., et al. (2008). Niche-specificity and the variable fraction of the Pectobacterium pan-genome. *Molecular plant-microbe interactions* 21(12)**,** 1549-1560.

Heeb, S., Itoh, Y., Nishijyo, T., Schnider, U., Keel, C., Wade, J., et al. (2000). Small, stable shuttle vectors based on the minimal pVS1 replicon for use in gram-negative, plant-associated bacteria. *Molecular Plant-Microbe Interactions* 13(2)**,** 232-237.

Katashkina, J.I., Hara, Y., Golubeva, L.I., Andreeva, I.G., Kuvaeva, T.M., and Mashko, S.V. (2009). Use of the λ Red-recombineering method for genetic engineering of Pantoea ananatis. *BMC molecular biology* 10(1)**,** 34.

Lagendijk, E.L., Validov, S., Lamers, G.E., De Weert, S., and Bloemberg, G.V. (2010). Genetic tools for tagging Gram-negative bacteria with mCherry for visualization in vitro and in natural habitats, biofilm and pathogenicity studies. *FEMS microbiology letters* 305(1)**,** 81-90.

Moleleki, L.N., Pretorius, R.G., Tanui, C.K., Mosina, G., and Theron, J. (2017). A quorum sensing-defective mutant of Pectobacterium carotovorum ssp. brasiliense 1692 is attenuated in virulence and unable to occlude xylem tissue of susceptible potato plant stems. *Mol Plant Pathol* 18(1)**,** 32-44. doi: 10.1111/mpp.12372.

Tanui, C.K., Shyntum, D.Y., Priem, S.L., Theron, J., and Moleleki, L.N. (2017). Influence of the ferric uptake regulator (Fur) protein on pathogenicity in Pectobacterium carotovorum subsp. brasiliense. *PLoS One* 12(5)**,** e0177647. doi: 10.1371/journal.pone.0177647.
